# Fragile X mental retardation protein regulates trans-synaptic signaling in *Drosophila*

**DOI:** 10.1242/dmm.012229

**Published:** 2013-09-05

**Authors:** Samuel H. Friedman, Neil Dani, Emma Rushton, Kendal Broadie

**Affiliations:** 1 Department of Biological Sciences, Kennedy Center for Research on Human Development, Vanderbilt University, Nashville, TN 37212, USA

## Abstract

Fragile X syndrome (FXS), the most common inherited determinant of intellectual disability and autism spectrum disorders, is caused by loss of the fragile X mental retardation 1 (*FMR1*) gene product (FMRP), an mRNA-binding translational repressor. A number of conserved FMRP targets have been identified in the well-characterized *Drosophila* FXS disease model, but FMRP is highly pleiotropic in function and the full spectrum of FMRP targets has yet to be revealed. In this study, screens for upregulated neural proteins in *Drosophila fmr1* (*dfmr1*) null mutants reveal strong elevation of two synaptic heparan sulfate proteoglycans (HSPGs): GPI-anchored glypican Dally-like protein (Dlp) and transmembrane Syndecan (Sdc). Our recent work has shown that Dlp and Sdc act as co-receptors regulating extracellular ligands upstream of intracellular signal transduction in multiple trans-synaptic pathways that drive synaptogenesis. Consistently, *dfmr1* null synapses exhibit altered WNT signaling, with changes in both Wingless (Wg) ligand abundance and downstream Frizzled-2 (Fz2) receptor C-terminal nuclear import. Similarly, a parallel anterograde signaling ligand, Jelly belly (Jeb), and downstream ERK phosphorylation (dpERK) are depressed at *dfmr1* null synapses. In contrast, the retrograde BMP ligand Glass bottom boat (Gbb) and downstream signaling via phosphorylation of the transcription factor MAD (pMAD) seem not to be affected. To determine whether HSPG upregulation is causative for synaptogenic defects, HSPGs were genetically reduced to control levels in the *dfmr1* null background. HSPG correction restored both (1) Wg and Jeb trans-synaptic signaling, and (2) synaptic architecture and transmission strength back to wild-type levels. Taken together, these data suggest that FMRP negatively regulates HSPG co-receptors controlling trans-synaptic signaling during synaptogenesis, and that loss of this regulation causes synaptic structure and function defects characterizing the FXS disease state.

## INTRODUCTION

Fragile X syndrome (FXS) is caused solely by loss of fragile X mental retardation protein (FMRP), which binds mRNAs to mediate transcript stability and trafficking, and acts as a negative regulator of translation (Laggerbauer et al., 2001; Li et al., 2001; Zhang et al., 2001; Lu et al., 2004; Muddashetty et al., 2007; Tessier and Broadie, 2008). Both humans with FXS and animal models of the disease (murine and *Drosophila*) exhibit synaptogenesis defects characterized by overgrowth and supernumerary synaptic contacts (Rudelli et al., 1985; Hinton et al., 1991; Gatto and Broadie, 2011; Tessier and Broadie, 2012). FXS disease models also exhibit defects in synaptic function, including elevated neurotransmission and altered activity-dependent plasticity (Zhang et al., 2001; Repicky and Broadie, 2009; Callan and Zarnescu, 2011; Gross et al., 2012). In the *Drosophila* FXS model, synaptic defects are rescued by introduction of human *FMR1*, but not the closely related *FXR1* or *FXR2* (Coffee et al., 2010; Tessier and Broadie, 2012), showing functional conservation of FMRP-dependent synaptogenic mechanisms. The numerous presynaptic and postsynaptic defects in the FXS disease state, which have often been first characterized in the *Drosophila* FXS model (Zhang et al., 2001; Pan et al., 2004; Pan and Broadie, 2007; Tessier and Broadie, 2012), have established clear roles for FMRP on both sides of the synaptic cleft. Conserved FMRP targets that have been functionally evaluated include presynaptic microtubule-associated protein 1B (MAP1B) (Zhang et al., 2001; Lu et al., 2004) and membrane-associated scaffold postsynaptic density protein of 95 kDa (PSD-95) (Zalfa et al., 2007; Muddashetty et al., 2011). Yet, the full spectrum of FMRP targets is unknown, and *Drosophila* remains an excellent model in which to study this complex regulation. Importantly, although some synaptogenic defects are rescued cell-autonomously, others require FMRP in the synaptic partner, demonstrating non-cell-autonomous requirements (Gatto and Broadie, 2008; Tessier and Broadie, 2012). Thus, FMRP might influence synaptogenesis via trans-synaptic signaling, regulating the cooperative differentiation of both sides of the synapse.

Trans-synaptic signaling pathways have been particularly well characterized at the *Drosophila* neuromuscular junction (NMJ) model synapse (Bayat et al., 2011; Dani and Broadie, 2012; Koles and Budnik, 2012; Rohrbough et al., 2013). A classic WNT pathway involves presynaptic secretion of Wingless (Wg), anterograde activation of postsynaptic Frizzled-2 (Fz2) receptors, internalization and cleavage of the Fz2 C-terminus (Fz2-C), and finally Fz2-C nuclear import leading to modulation of synaptic structure and function (Packard et al., 2003; Salinas, 2003; Mathew et al., 2005; Koles and Budnik, 2012). Recent work has shown that Fz2-C localizes with translationally silenced ribonucleoprotein particles and aids in their trafficking outside the nucleus, facilitating local protein synthesis (Speese et al., 2012). A BMP pathway involves postsynaptic secretion of Glass bottom boat (Gbb), retrograde activation of presynaptic receptors containing Wishful thinking (Wit) and phosphorylation of the Mothers against decapentaplegic (MAD) transcription factor to similarly modulate synaptic structure and function (McCabe et al., 2003; Keshishian and Kim, 2004; Marqués, 2005; Nahm et al., 2010; Bayat et al., 2011). In addition to these two classic trans-synaptic pathways (Wg and Gbb), we recently discovered a new anterograde pathway involving presynaptic secretion of Jelly belly (Jeb) ligand to activate postsynaptic Anaplastic lymphoma kinase (Alk) receptors and drive phosphorylation of ERK (dpERK), resulting again in modulation of both synaptic structure and function (Rohrbough and Broadie, 2010; Rohrbough et al., 2013). In this study, we began with the hypothesis that FMRP non-cell-autonomous roles might occur via misregulation of one or more of these trans-synaptic signaling pathways.

**TRANSLATIONAL IMPACT**

**Clinical issue**

Fragile X syndrome (FXS), which occurs in about 1 in 4000 men and 1 in 6000 women, presents with a wide spectrum of neurodevelopmental deficiencies, including learning and memory impairments, hyperactivity and childhood seizures, and autism spectrum behaviors. These symptoms arise solely from the loss of the fragile x mental retardation 1 (*FMR1*) gene product FMRP, an RNA-binding translational regulator. Pre- and postsynaptic defects, characterized by structural abnormalities and altered neurotransmission, and defects in activity-dependent synaptic plasticity underlie the symptoms of FXS. Because trans-synaptic intercellular signaling is a key feature of synaptic development and plasticity – it controls the operative interplay orchestrating synaptic connectivity and communication strength – defects in trans-synaptic signaling might therefore be involved in the development of the coupled preand postsynaptic deficits seen in the FXS disease state.

**Results**

In this study, the authors use the well-characterized *Drosophila* FXS disease model (*dfmr1* null) to test whether misregulation of known trans-synaptic signaling pathways is a central feature of the FXS disease state. Using a candidate screening approach, they show that two synaptic heparan sulfate proteoglycans (HSPGs) that act as extracellular co-receptors for WNT and BMP intercellular signaling ligands are strongly upregulated in *dfmr1* null flies. Examination of the anterograde WNT and retrograde BMP trans-synaptic signaling cascades and the recently identified anterograde Jeb-Alk signaling pathway at the neuromuscular junction model synapse indicates that both the WNT and the Jeb-Alk trans-synaptic pathways are strongly misregulated in *dfmr1* null flies but that the BMP pathway is unaltered. Finally, the authors show that correction of HSPG upregulation is sufficient to fully restore both synaptic architecture and neurotransmission strength to the wild-type condition.

**Implications and future directions**

These findings show that key synaptogenic HSPGs are upregulated and trans-synaptic signaling pathways are strongly impaired in the absence of FMRP, thereby identifying trans-synaptic signaling misregulation as a central feature of the FXS disease state, at least in the *Drosophila* genetic model. Recent promising clinical trials on potential treatments for FXS that are founded on insights from animal disease models have focused attention on extracellular synaptic components and the mechanisms of trans-synaptic signaling. Moreover, current FDA-approved therapies for FXS, such as lithium, have nonlinear activities that involve components of the trans-synaptic signaling pathway identified here. Thus, taken together with existing clinical experience, these findings strongly suggest that further investigation into trans-synaptic signaling has the potential to identify additional therapeutic avenues for the treatment of FXS.

As the candidate coupling mechanism, three independent lines of investigation converged to focus our attention on heparan sulfate proteoglycans (HSPGs), a class of membrane-bound proteins with two or three glycosaminoglycan chains composed of a repeating sulfated disaccharide. These repeats, which are in close proximity to the cell surface, bind protein signaling ligands (Dani and Broadie, 2012). First, it was recently shown in both *Drosophila* and mouse FXS models that FMRP regulates extracellular matrix metalloproteinases (MMPs) in a pathway that is central to the control of synaptic properties (Bilousova et al., 2009; Siller and Broadie, 2011). In *Drosophila*, we showed that *dfmr1* null synaptogenic defects are effectively prevented by pharmacological inhibition of MMP activity, transgenic overexpression of the endogenous tissue inhibitor of MMPs (TIMP), or co-removal of secreted MMP-1 (Siller and Broadie, 2011; Siller and Broadie, 2012). Importantly, HSPGs are well-established MMP proteolytic targets (d’Ortho et al., 1997; Yu and Woessner, 2000; Egeblad and Werb, 2002; Choi et al., 2012). Moreover, two synaptic HSPGs at the *Drosophila* NMJ, GPI-anchored Dally-like protein (Dlp) and transmembrane Syndecan (Sdc), play key roles in the modulation of synaptic structure and function (Johnson et al., 2006). Second, we have recently shown that misregulation of Dlp and/or Sdc HSPGs is causative in multiple trans-synaptic signaling defects at the *Drosophila* NMJ (Dani et al., 2012). Specifically, we demonstrated that Dlp and Sdc act as co-receptors for extracellular ligands to regulate their abundance and modulate downstream signaling. Additionally, we revealed that regulation of the extracellular ligands is interdependent on the relative abundance of both cognate receptor and HSPG co-receptor, creating the tiered system of regulation postulated in the ‘exchange-factor model’ (Yan et al., 2009; Dani et al., 2012). Finally, HSPG RNA transcripts are direct binding targets of FMRP, as recently shown by high-throughput sequencing of RNA isolated via crosslinking immunoprecipitation (HITS-CLIP) (Darnell et al., 2011). Taken together, these lines of evidence support the hypothesis that synaptic levels might be altered in the *dfmr1* null disease state to cause impaired trans-synaptic signaling and thus defects in synaptic structure and function.

To test this hypothesis, we first assayed the synaptic expression of Dlp and Sdc HSPGs, and found that both were highly elevated in *dfmr1* null NMJ synapses, consistent with FMRP functioning as a translational repressor. We then tested each of the above three trans-synaptic pathways (Wg, Gbb and Jeb), and found that both anterograde pathways (Wg and Jeb) were strongly dysregulated at *dfmr1* null synapses, but there was no change in the retrograde pathway (Gbb). The ‘exchange-factor model’ predicts that downstream signaling is dependent on the ratio of Wg ligand, Dlp co-receptor and Fz2 receptor, such that excess Dlp causes Wg to be sequestered away from Fz-2 receptors, causing Wg accumulation without activating downstream signaling. Consistent with this prediction, the changes in the downstream signaling of Fz2-C (Wg pathway) and dpERK (Jeb pathway) occurred proportionally to changes in the ratio of ligand (Wg and Jeb) versus HSPG co-receptor (Dlp and Sdc) at *dfmr1* null synapses, whereas pMAD signaling (Gbb pathway) was not altered. Mechanistically, HSPG overexpression mimicked *dfmr1* null synaptic phenotypes, and genetically correcting HSPG elevation in the *dfmr1* null background restored both excess synaptic structure and elevated neurotransmission strength back to wild-type levels. Importantly, genetically correcting HSPG elevation in the *dfmr1* null background was sufficient to restore normal trans-synaptic signaling. Taken together, these results suggest that FMRP repression of synaptic HSPG co-receptors normally regulates trans-synaptic signaling to modulate synaptic structure and function, and that disruption of this mechanism is causal in synaptogenesis defects in the *Drosophila* disease model of fragile X syndrome.

## RESULTS

### Two synaptic HSPGs are strongly upregulated in the absence of FMRP

**Fig. 1.**
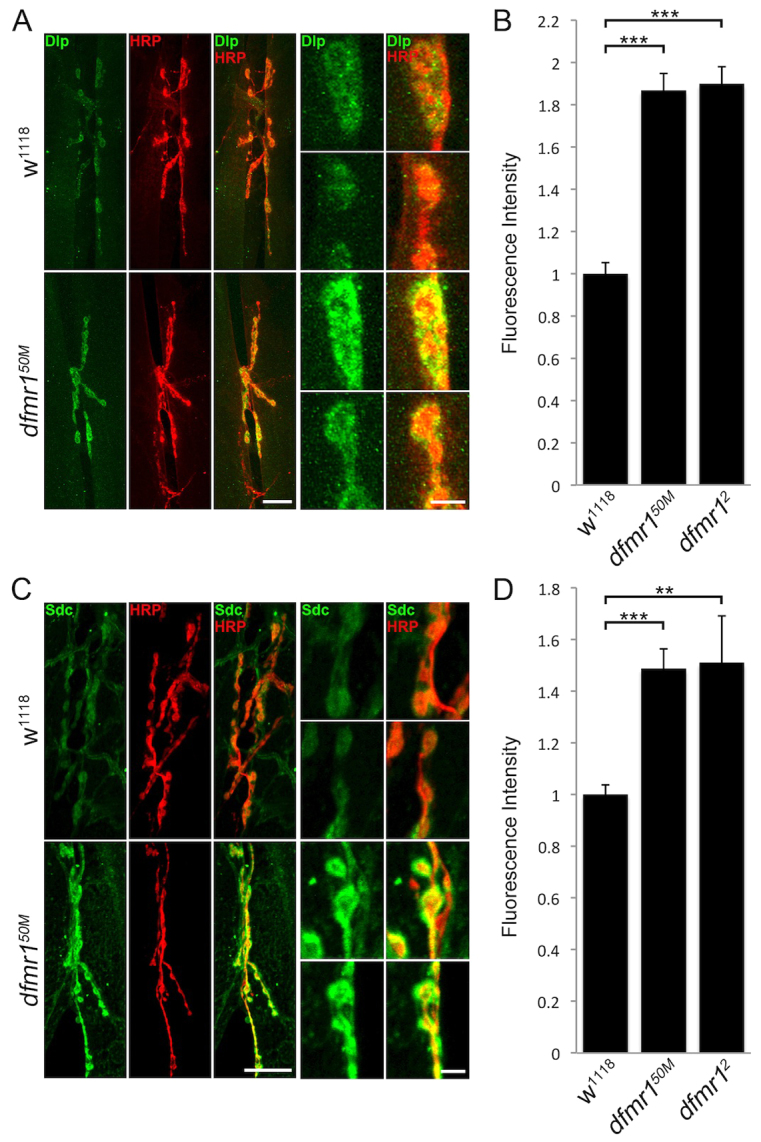
**Highly elevated HSPG co-receptor levels at *dfmr1* null NMJs.** (A) Representative NMJ images co-labeled with neuronal marker anti-horseradish peroxidase (HRP, red) and anti-Dlp (Dlp, green) in control (*w^1118^*) and *dfmr1* null (*dfmr1^50M^*) wandering third instar muscle 6/7. Right panels show synaptic boutons in higher magnification. Scale bars: 25 μm and 5 μm, respectively. (B) Quantification of Dlp intensity normalized to genetic controls (*w^1118^*) in two *dfmr1* null mutants (*dfmr1^50M^*, *dfmr1^2^*). Sample sizes are ≥12 animals and ≥24 NMJs for each genotype. (C) Representative NMJ images co-labeled with neuronal marker (HRP, red) and anti-Sdc (Sdc, green) in *w^1118^* and *dfmr1^50M^* flies. Scale bars: 25 μm and 5 μm (higher magnification). (D) Quantification of Sdc intensity levels. Sample sizes are ≥17 animals and ≥34 NMJs for each genotype. Significance is shown as **P<0.01 and ***P<0.001.

The extracellular synaptomatrix plays crucial roles in shaping *Drosophila* NMJ synaptic development and modulation (Broadie et al., 2011), with conserved functions in mammalian synapse structural and functional maturation (Dityatev and Schachner, 2006; Vautrin, 2010; Barros et al., 2011). For example, two membrane-anchored HSPGs regulate structural and functional differentiation of the *Drosophila* NMJ; GPI-anchored Dlp and transmembrane Sdc (Johnson et al., 2006). In other cellular contexts, these same HSPGs act as crucial regulators of intercellular communication (Yan and Lin, 2009; Kleinschmit et al., 2010; Dejima et al., 2011), and we have recently shown that these two HSPGs function as co-receptors controlling secreted ligand abundance during trans-synaptic signaling at the *Drosophila* NMJ (Dani et al., 2012). Given that HSPG transcripts were recently identified as FMRP direct-binding targets by HITS-CLIP (Darnell et al., 2011), we hypothesized that changes in HSPG protein levels at the synapse could provide a candidate mechanism to explain synaptogenesis defects in the *dfmr1* null disease model (Zhang et al., 2001; Pan et al., 2004; Gatto and Broadie, 2008). To begin to test this hypothesis, we first probed Dlp and Sdc expression at the NMJ using well-characterized antibodies (Fig. 1).

In genetic controls (*w^1118^*) and two *dfmr1* null mutants, we analyzed NMJs of mature larvae for Dlp and Sdc, co-labeling with anti-horseradish peroxidase (HRP) to mark neuronal membranes (Fig. 1). In controls, Dlp exhibited punctate expression surrounding each HRP-marked presynaptic bouton (Fig. 1A). Dlp seemed to be specifically localized at boutons, with axonal expression largely undetectable. In contrast, *dfmr1* null NMJs showed a clear increase in Dlp expression intensity, with a divergence from control punctate expression towards a pattern that covers most of the bouton (Fig. 1A). Dlp expression was quantified by measuring fluorescence intensity within the HRP-marked region to define the synaptic domain. Comparative measurements show that Dlp expression was elevated by ∼90% in two *dfmr1* nulls compared with controls (fluorescence intensity: normalized *w^1118^*, 1.0±0.044; *dfmr1^50M^*, 1.90±0.074, *P*<0.001; *dfmr1^2^*, 1.88±0.070, *P*<0.001; Fig. 1B). Similarly, in control NMJs, Sdc exhibited a halo-like expression pattern surrounding HRP-marked synaptic boutons (Fig. 1C). Likewise, this HSPG was significantly elevated in abundance at *dfmr1* null synapses compared with in controls, exhibiting a more intense expression closely overlapping with the HRP signal (Fig. 1C). In quantifying intensity, Sdc expression was found to be increased by ∼50% over control levels (fluorescence intensity: *w^1118^*, 1.0±0.037; *dfmr1^50M^*, 1.49±0.076, *P*<0.001; *dfmr1^2^*, 0.18±0.06, *P*<0.01; Fig. 1D). These results show that both Dlp and Sdc HSPGs are strongly upregulated at the NMJ synapse in the absence of FMRP.

### Elevated abundance of the WNT ligand Wg in the synaptomatrix signaling domain

**Fig. 2.**
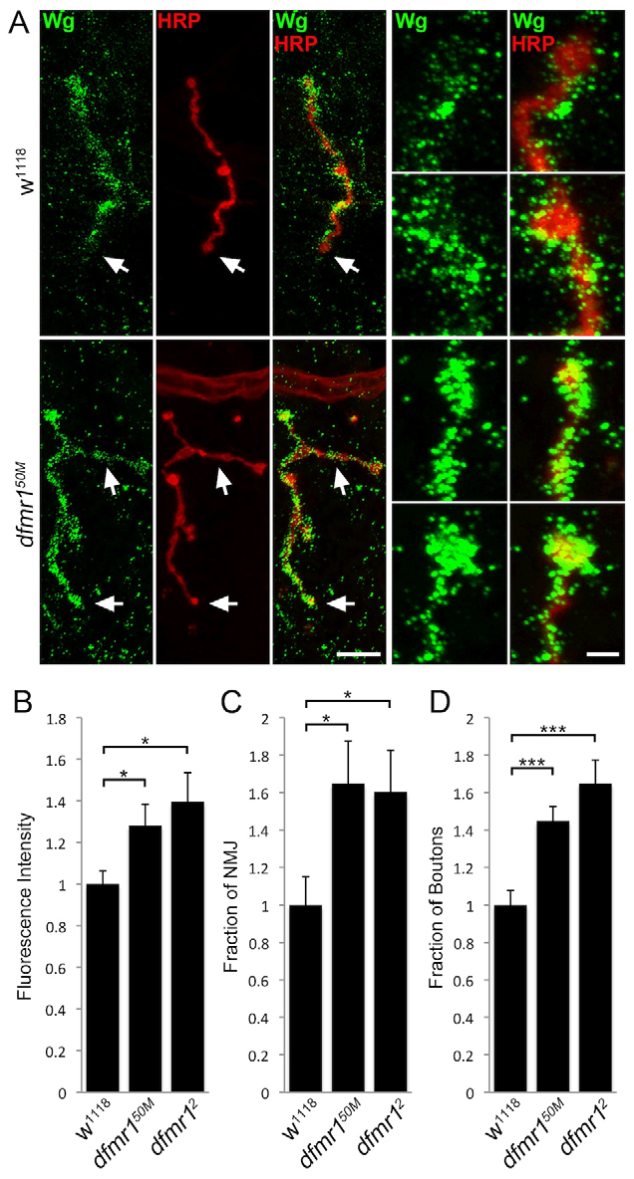
**Increased Wg levels and spatial distribution at *dfmr1* null NMJs.** (A) Representative NMJ images co-labeled with neuronal marker (HRP, red) and anti-Wingless (Wg, green) in control (*w^1118^*) and *dfmr1* null (*dfmr1^50M^*) wandering third instar muscle 4. Right panels show synaptic boutons at higher magnifications. Scale bars: 15 μm and 3 μm, respectively. Arrows indicate boutons that have been magnified. (B) Quantification of Wg intensity in *dfmr1* nulls normalized to *w^1118^* controls. (C) Analysis of Wg spatial distribution measured as a fraction of the total HRP-labeled NMJ area. (D) Fraction of total HRP-labeled NMJ boutons expressing Wg. Samples sizes are ≥26 animals and ≥52 NMJs for each genotype. Significance is shown as *P<0.05 and ***P<0.001.

We have recently shown that these membrane HSPGs act as coreceptors for the WNT ligand Wg at the *Drosophila* NMJ (Dani et al., 2012). They act to trap the Wg ligand at the synaptic interface, with regulation of HSPG sulfation state determining synaptic Wg levels (Dani et al., 2012). Wg is the best-characterized trans-synaptic signal in this system; it is secreted from the presynaptic terminal to activate postsynaptic receptors in an anterograde signaling pathway, as well as activating autocrine presynaptic receptors, to modulate both structural and functional synaptogenesis (Salinas, 2003; Koles and Budnik, 2012). Multiple studies show that Wg is regulated in both absolute abundance and spatial distribution by the Dlp HSPG in other *Drosophila* cellular contexts (Khare and Baumgartner, 2000; Kirkpatrick et al., 2004; Han et al., 2005; Kleinschmit et al., 2010; Wu et al., 2010). We therefore hypothesized that the strong upregulation of GPI-anchored Dlp in the NMJ synaptomatrix should drive parallel upregulation of Wg in the *dfmr1* null condition. To test this hypothesis, we first analyzed Wg ligand extracellular expression at NMJs of wandering third instar larvae, co-labeled with the presynaptic marker anti-HRP (Fig. 2).

All studies were performed in detergent-free, unpermeabilized conditions, to selectively visualize only extracellular Wg (Rushton et al., 2009; Dani et al., 2012). As discovered previously, we observed that Wg exhibits dynamic expression at the *Drosophila* NMJ, with strong expression in a fluctuating subset of synaptic boutons (Fig. 2A). In control *w^1118^* synapses, Wg appeared as a punctate pattern across the surface of HRP-marked boutons, with different boutons expressing different amounts of the signal. At *dfmr1* null NMJs, there was a clearly increased intensity of Wg expression, and a broader spatial distribution compared with controls (Fig. 2A). In quantifying Wg intensity, comparative measurements showed that *dfmr1* nulls displayed a significantly elevated overall abundance compared with controls (fluorescence intensity: normalized *w^1118^*, 1.0±0.062; *dfmr1^50M^*, 1.28±0.103; *dfmr1^2^*, 1.39±0.14, *P*<0.05; Fig. 2B). In addition, *dfmr1* nulls showed an increase in the spatial distribution of Wg, both as the fraction of NMJ area expressing Wg and as the number of synaptic boutons expressing Wg. We quantified these parameters by measuring Wg expression area and dividing by HRP-marked area, to normalize for NMJ size. Quantification showed that Wg area is ∼65% higher in *dfmr1* nulls compared with controls (fraction of NMJ area expressing Wg: *w^1118^*, 1.0±0.15; *dfmr1^50M^*, 1.65±0.23; *dfmr1^2^*, 1.60±0.22, *P*<0.05; Fig. 2C). We finally measured the number of Wg-positive synaptic boutons as a fraction of total boutons, showing that controls exhibit ∼50% Wg-positive boutons and *dfmr1* nulls ∼75% Wg-positive boutons (fraction of boutons expressing Wg: *w^1118^*, 1.0±0.076; *dfmr1^50M^*, 1.45±0.077; *dfmr1^2^*, 1.65±0.13, *P*<0.001; Fig. 2D). Taken together, these data show that *dfmr1* synapses exhibit increased Wg ligand abundance and spatial distribution.

### An increased ratio of HSPG co-receptor to Wg ligand depresses Fz2-C signaling

**Fig. 3.**
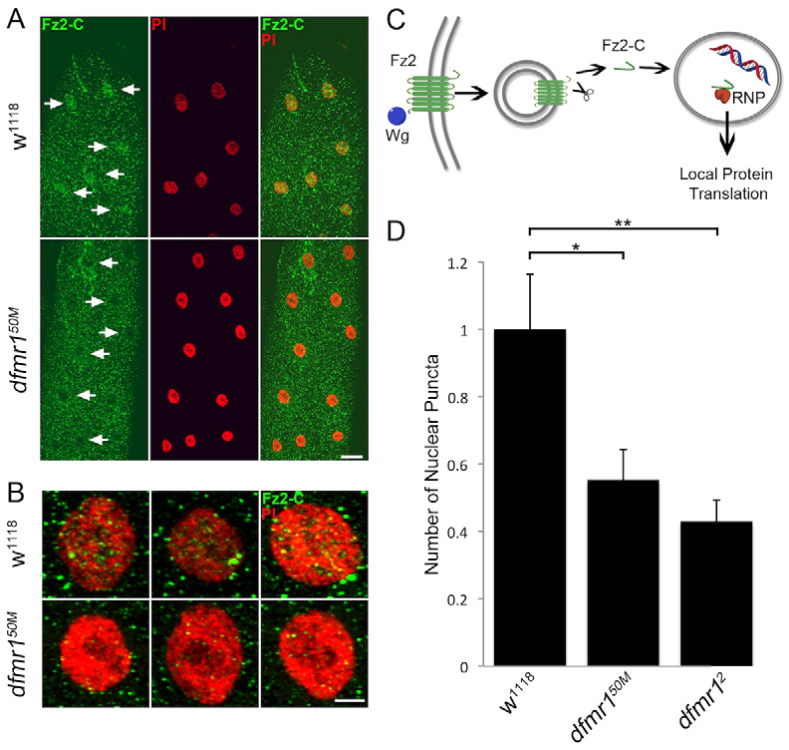
**Loss of Fz2 C-terminus nuclear translocation signaling at *dfmr1* null NMJs.** (A) Representative muscle 4 images co-labeled with nuclear marker propidium iodide (PI, red) and anti-Frizzled C-terminus (Fz2-C, green) in control (*w^1118^*) and *dfmr1* null (*dfmr1^50M^*) wandering third instars. Arrows indicate Fz2-C marked nuclei in controls. Scale bar: 20 μm. (B) Higher-magnification images of individual nuclei co-labeled with nuclear marker (PI, red) and anti-Fz2-C (green). Scale bar: 5 μm. (C) Schematic depicting the pathway of Wg binding to Fz2, resulting in postsynaptic nuclear localization of the receptor C-terminus. (D) Quantification of Fz2-C nuclear localization measured as Fz2-C puncta number in the nuclei, normalized to *w^1118^* genetic controls. Sample sizes are ≥7 animals and ≥14 muscles for each genotype. Significance is shown as *P<0.05 and **P<0.01.

Wg acts through the Fz2 nuclear import pathway (Mathew et al., 2005): Wg binding to postsynaptic Fz2 receptors stimulates receptor internalization and cleavage of the C-terminus (Fz2-C), which translocates to postsynaptic nuclei to control ribonucleoprotein (RNP) export and local translation (Fig. 3) (Speese et al., 2012). Regulation of this signaling pathway is quantified by counting the number of Fz2-C RNP granule puncta in muscle nuclei (Mathew et al., 2005; Speese et al., 2012). Using this measure, we have recently shown that HSPG modulation regulates Wg abundance at the NMJ synapse to control Fz2-C nuclear translocation (Dani et al., 2012). The exchange-factor model states that downstream signaling is dependent on the ratio of Wg ligand, Dlp co-receptor and Fz2 receptor, such that excess Dlp causes Wg to be sequestered away from Fz-2 receptors, causing Wg accumulation without activating downstream signaling (Yan et al., 2009). Based on this foundation, it is possible that there could be a similar downstream impact on the Fz2-C nuclear import pathway in the *dfmr1* null condition. We tested this hypothesis by examining Fz2-C expression in postsynaptic muscle nuclei (Fig. 3).

Wandering third instar muscles were co-labeled with anti-Fz2-C and the nuclear marker propidium iodide (PI; Fig. 3A). Strikingly, in low-magnification images of muscle, Fz2-C nuclear accumulation was clearly detectable in genetic controls (Fig. 3A, arrows), yet obviously absent in *dfmr1* null mutants (Fig. 3A). High-magnification images of control nuclei show clear punctate domains within the PI-marked nucleus, indicating an accumulation of Fz2-C RNPs (Fig. 3B). There is also a build-up of Fz2-C puncta directly surrounding the nucleus in controls, presumably representing Fz2-C being trafficked to/from the nucleus (Speese et al., 2012). In *dfmr1* nulls, the amounts of both the intra-nuclear and extra-nuclear puncta were very markedly reduced (Fig. 3B), denoting a decrease in the activity of the intracellular signaling cascade (Fig. 3C). To quantify this pathway, we counted the number of Fz2-C puncta in nuclei for both control and *dfmr1* null mutants, and then calculated an average number of puncta per nucleus normalized for each muscle (Fig. 3D). These analyses revealed a stark decrease in Fz2-C nuclear localization in *dfmr1* nulls compared with controls (normalized *w^1118^*, 1.0±0.16; *dfmr1^50M^*, 0.55±0.09, *P*<0.05; *dfmr1^2^*, 0.43±0.06, *P*<0.01; Fig. 3D). These results show that, despite increased Wg abundance in the extracellular synaptomatrix, the Fz2 nuclear import signaling pathway is downregulated in the absence of FMRP. Our interpretation of this result, as we have recently shown (Dani et al., 2012), is that dramatic overexpression of HSPG co-receptors sequesters Wg ligand away from Fz2 receptors, and thereby depresses overall Wg trans-synaptic signaling in the *dfmr1* null condition.

### A second anterograde trans-synaptic pathway is depressed at *dfmr1* null NMJs

**Fig. 4.**
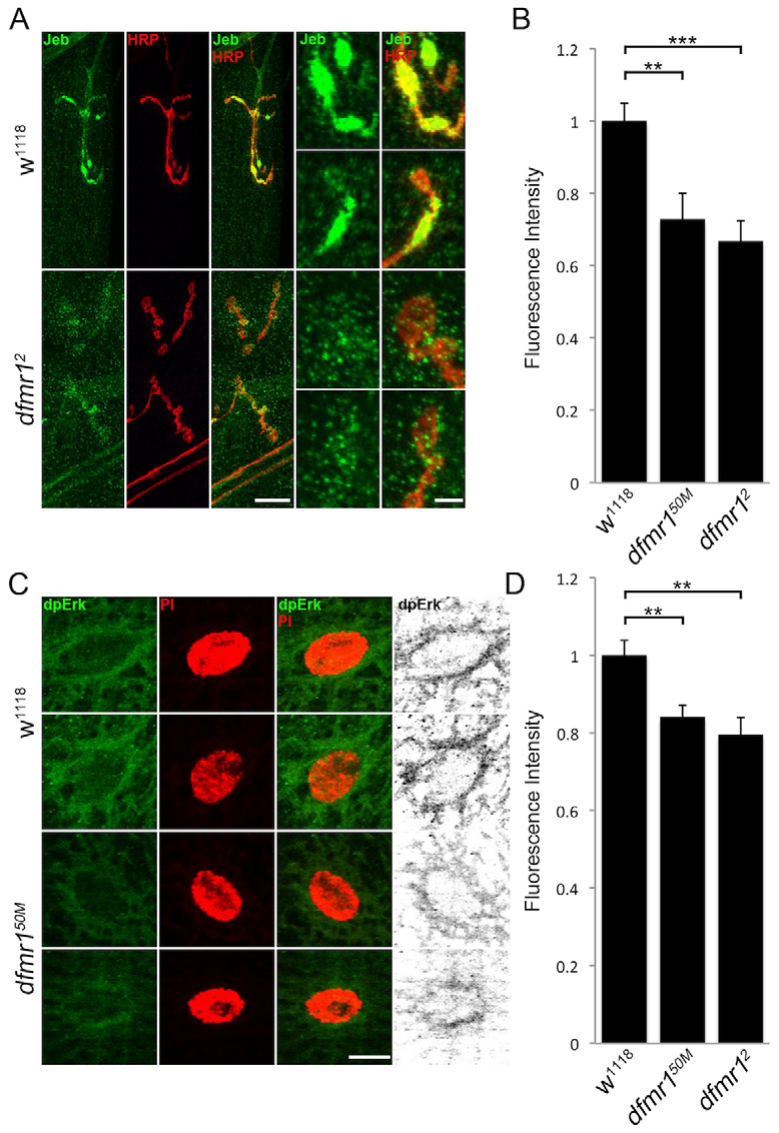
**Reduced Jeb ligand levels and dpERK signaling at *dfmr1* null NMJs.** (A) Representative NMJ images co-labeled with neuronal marker (HRP, red) and anti-Jeb (green) in control (*w^1118^*) and *dfmr1* null (*dfmr1^2^*) wandering third instar muscle 4. Right panels show synaptic boutons at higher magnification. Scale bars: 15 μm and 5 μm, respectively. (B) Quantification of Jeb intensity in two *dfmr1* null alleles (*dfmr1^50M^*, *dfmr1^2^*), normalized to genetic controls (*w^1118^*). Sample sizes are ≥9 animals and ≥18 NMJs. (C) Representative images of individual muscle nuclei co-labeled with nuclear marker (PI, red) and anti-diphosphorylated extracellular signal regulated kinase (dpERK, green) in *w^1118^* and *dfmr1^50M^*. Scale bar: 10 μm. (D) Quantification of dpERK intensity in two *dfmr1* nulls (*dfmr1^50M^*, *dfmr1^2^*), normalized to *w^1118^*. Sample sizes ≥8 animals, ≥16 NMJs. Significance shown as **P<0.01 and ***P<0.001.

We have recently established Jeb as another presynaptically secreted signaling ligand, which binds postsynaptic Alk receptors to activate an anterograde MAPK pathway of ERK phosphorylation (dpERK) and nuclear import (Rohrbough and Broadie, 2010; Rohrbough et al., 2013). Although Jeb has not been shown to interact with an HSPG co-receptor, it is known to be regulated by the endogenous lectin Mind-the-gap (MTG) (Rohrbough and Broadie, 2010). Given the strong effect on Wg trans-synaptic signaling in the *dfmr1* null disease state, we wished to next test specificity by determining whether this separate anterograde pathway might also be impacted. We therefore performed similar experiments, probing the NMJ synapse with well-characterized antibodies to Jeb (Englund et al., 2003) and activated dpERK (Yung et al., 1997), with the NMJ co-labeled with HRP and the PI nuclear markers, respectively. We assayed Jeb expression with detergent-free labeling, as above using unpermeabilized conditions to selectively visualize the extracellular ligand (Rushton et al., 2009). A summary of these data are shown in Fig. 4.

In genetic controls (*w^1118^*), Jeb expression was strongly associated with NMJ boutons, closely overlapping with the HRP-marked neuronal membrane, with little or no detectable expression associated with axonal regions (Fig. 4A). In *dfmr1* null NMJs, Jeb expression was very strongly reduced. In mutants, Jeb seems to maintain bouton-specific localization, yet the boutons secrete less detectable ligand (Fig. 4A). Fluorescence intensity quantification shows a highly significant loss of Jeb in *dfmr1* nulls compared with controls (normalized *w^1118^*, 1.0±0.049; *dfmr1^50M^*, 0.73±0.071, *P*<0.01; *dfmr1^2^*, 0.67±0.056, *P*<0.001; Fig. 4B). Jeb on the extrasynaptic muscle surface, away from the NMJ terminal, was similarly reduced (*w^1118^*, 1.0±0.049; *dfmr1^50M^*, 0.73±0.071, *P*<0.01; *dfmr1^2^*, 0.67±0.056, *P*<0.001; supplementary material Fig. S1). In parallel, we analyzed downstream dpERK signaling in muscle nuclei. In controls, dpERK was expressed in NMJs and within postsynaptic muscle nuclei (Fig. 4C). In *dfmr1* nulls, the amount of activated dpERK trafficked to muscle nuclei was obviously reduced. In mutants, dpERK spatial localization was similar to controls, but fluorescence intensity is clearly diminished. We quantified dpERK labeling within PI-marked muscle nuclei (Fig. 4D). In the *dfmr1* null condition, dpERK nuclear localization was significantly reduced compared with controls (fluorescence intensity: *w^1118^*, 1.0±0.04; *dfmr1^50M^*, 0.84±0.03, *P*<0.05; *dfmr1^2^*, 0.80±0.04, *P*<0.05). These results show that both Wg–Fz2-C and Jeb-dpERK anterograde signaling is decreased in the absence of FMRP.

### Retrograde BMP signaling is not altered at *dfmr1* null NMJs

**Fig. 5.**
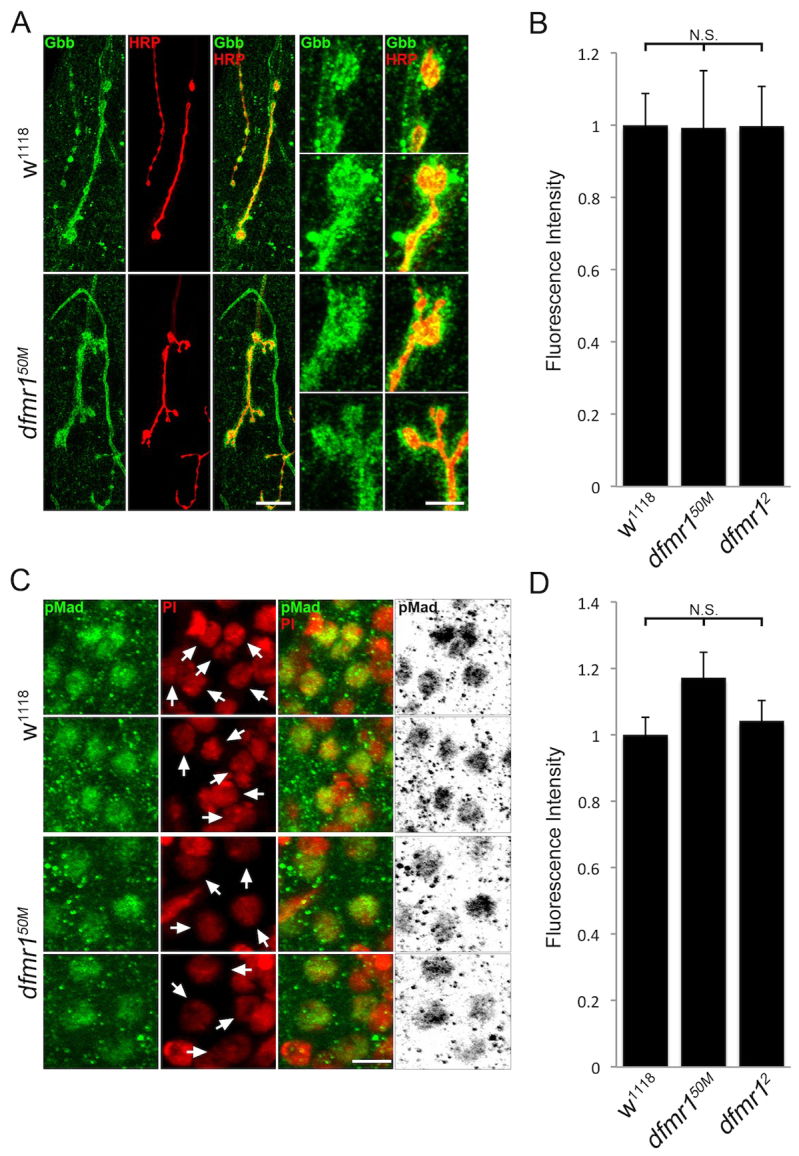
**No detectable change in retrograde BMP signaling at *dfmr1* null NMJs.** (A) Representative NMJ images co-labeled with neuronal marker (HRP, red) and anti-Gbb (green) in control (*w^1118^*) and *dfmr1* null (*dfmr1^50M^*) wandering third instar muscle 4. Right panels show synaptic boutons at higher magnification. Scale bars: 15 μm and 5 μm, respectively. (B) Quantification of Gbb intensity in two *dfmr1* nulls (*dfmr1^50M^*, *dfmr1^2^*), normalized to *w^1118^* genetic controls. Sample size is ≥8 animals and ≥16 NMJs for each genotype. (C) Representative images of motor neuron nuclei in the larval CNS co-labeled with nuclear marker (PI, red) and anti-pMAD (green) in *w^1118^* and *dfmr1^50M^*. Arrows indicate motor neuron nuclei. Black/white images are shown to better highlight differences. Scale bar: 5 μm. (D) Quantification of pMAD intensity in two *dfmr1* nulls (*dfmr1^50M^*, *dfmr1^2^*), normalized to *w^1118^*. Sample size is ≥15 animals and ≥30 NMJs for each genotype. Statistical significance is shown as N.S. (*P*>0.05).

Given that two anterograde trans-synaptic pathways are misregulated in parallel in the absence of FMRP, we next set out to test whether retrograde signaling is similarly impacted. A well-characterized retrograde pathway involves postsynaptic secretion of the BMP Gbb, which binds presynaptic receptors to stimulate downstream phosphorylation of the transcription factor MAD (pMAD), which translocates to motor neuron nuclei in the CNS (McCabe et al., 2003; Keshishian and Kim, 2004; Kim and Marqués, 2010). Using an anti-Gbb antibody that we recently characterized (Dani et al., 2012), Gbb levels were compared between genetic control (*w^1118^*) and *dfmr1* null synapses. In parallel, we used a widely employed anti-pMAD antibody (Persson et al., 1998) to assay the downstream signal transduction pathway. A summary of these studies is shown in Fig. 5.

Detergent-free conditions were again employed to visualize only secreted Gbb at HRP-labeled NMJs (Fig. 5A). In controls, Gbb exhibited consistent high expression surrounding synaptic terminals, with Gbb expression surrounding and extending beyond HRP-marked synaptic boutons. In qualitative comparisons, *dfmr1* null Gbb expression seemed very similar to controls (Fig. 5A). Quantified Gbb intensity analyses similarly revealed no detectable changes in Gbb levels at *dfmr1* null NMJs compared with controls (normalized *w^1118^*, 1.0±0.087; *dfmr1^50M^*, 0.99±0.16, *P*>0.05; *dfmr1^2^*, 1.0±0.11, *P*>0.05; Fig. 5B). Although the Gbb abundance was not detectably changed in *dfmr1* nulls, we still investigated downstream pMAD signaling to test for possible functional misregulation. Because activated pMAD translocates into presynaptic nuclei, we measured signaling by imaging motor neuron nuclei in the CNS (Fig. 5C). PI was again used to mark nuclei (Fig. 5C, red), showing non-pMAD interneuron nuclei adjacent to pMAD-positive motor neuron nuclei (Fig. 5C, green; yellow overlap). We quantified pMAD expression intensity by outlining motor neuron nuclei in the PI red channel and measuring pMAD intensity in the green channel. This quantification revealed no detectable pMAD changes in *dfmr1* nulls compared with controls (*w^1118^*, 1.0±0.052; *dfmr1^50M^*, 1.17±0.077, *P*>0.05; *dfmr1^2^*, 1.04±0.061, *P*>0.05; Fig. 5D). Thus, we conclude that the retrograde BMP pathway is independent of FMRP regulation, suggesting that FMRP selectively regulates anterograde trans-synaptic signaling.

### Genetically reducing HSPG levels in *dfmr1* nulls restores synaptic architecture

**Fig. 6.**
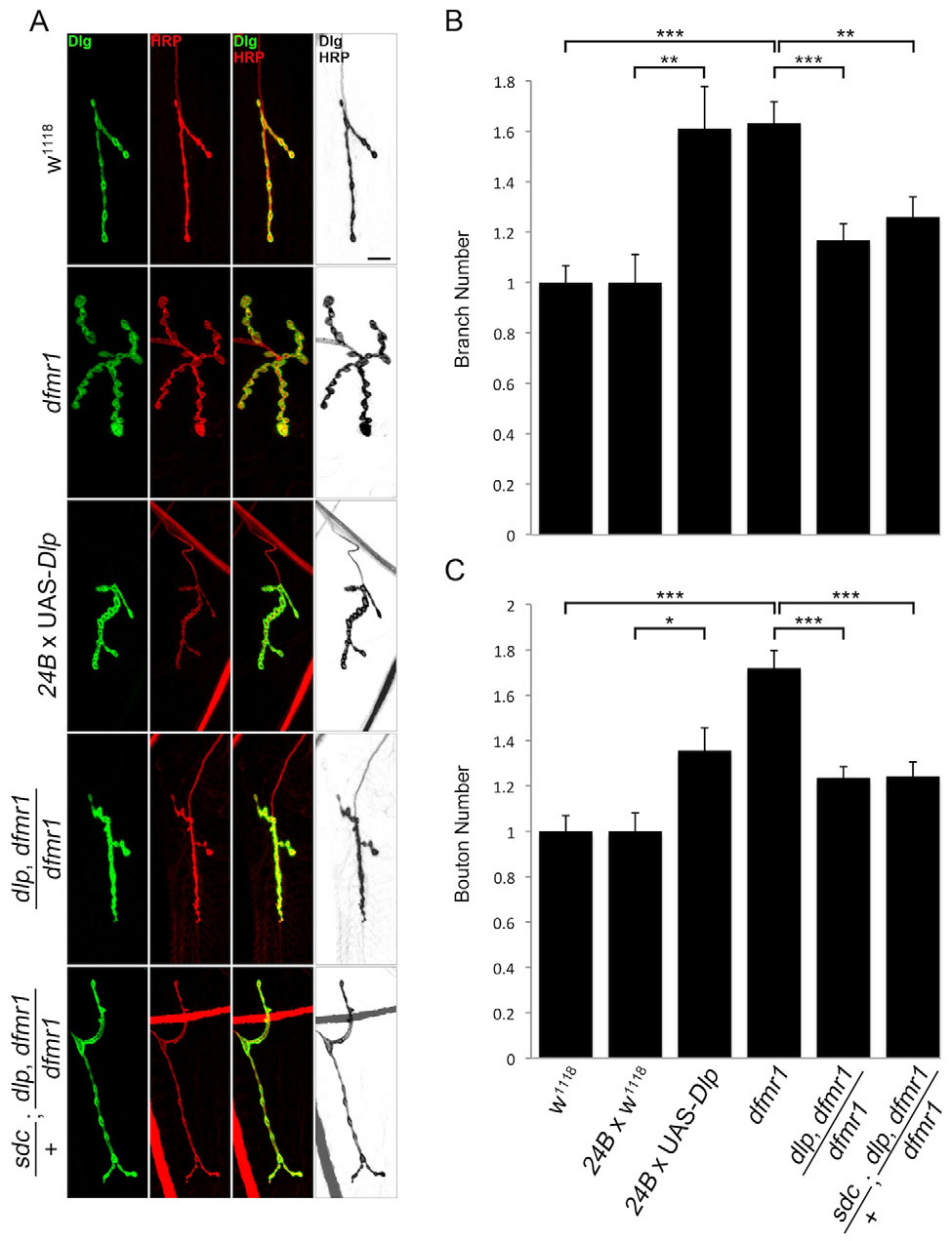
**Restoration of *dfmr1* null synaptic architecture defects by HSPG reduction.** (A) Representative muscle 4 NMJ images co-labeled with presynaptic marker anti-HRP (red) and postsynaptic marker anti-Discs-large (DLG, green) in control (*w^1118^*), *dfmr1*-null (*dfmr1^50M^*), postsynaptic Dlp overexpression (*24B* × UAS-*Dlp*), genetic reduction of Dlp in *dfmr1* null background (*dlp^A18^*, *dfmr1^50M^*/*dfmr1^50M^*), and genetic reduction of both Sdc and Dlp in *dfmr1* null background (*sdc^23^*/+; *dlp^A187^*, *dfmr1^50M^*/ *dfmr1^50M^*). Black/white images are shown to better highlight differences. Scale bar: 10 μm. (B,C) Quantification of total NMJ branches (B) and type 1 bouton number (C) in *dfmr1* null, Dlp overexpression, and HSPG genetic reductions conditions, normalized to genetic controls. Sample size is ≥4 animals and ≥8 NMJs for each indicated genotype. Significance is shown as *P<0.05, **P<0.01 and ***P<0.001.

The overelaboration of NMJ architecture caused by *dfmr1* loss has been well documented, including observations of increased axonal branching and supernumerary bouton formation (Zhang et al., 2001; Gatto and Broadie, 2008). Likewise, Wg and Jeb trans-synaptic signals have previously been well documented to similarly regulate synaptic architecture (McCabe et al., 2003; Korkut and Budnik, 2009; Ball et al., 2010; Budnik and Salinas, 2011; Rohrbough et al., 2013). We therefore hypothesized that FMRP-dependent regulation of trans-synaptic signaling could contribute to *dfmr1* structural phenotypes. Moreover, we hypothesized the causal link between FMRP and trans-synaptic signaling to be the HSPG co-receptors, which we have recently shown control trans-synaptic signaling to modulate NMJ structure (Dani et al., 2012). If this model is correct, then correcting HSPG levels in *dfmr1* nulls should restore synaptic architecture back towards wild-type levels. To test this hypothesis, we crossed heterozygous *dlp*/+ and *sdc*/+ null mutations into *dfmr1* homozygous null backgrounds, both singly and in combination, and assayed synaptic branching and bouton number in the resulting triple-mutant animals. A summary of these studies is shown in Fig. 6.

We assayed NMJ structure by co-labeling with anti-HRP and anti-DLG (Discs-large) to delineate the pre- and postsynaptic terminals, respectively (Fig. 6A). Type 1 synaptic boutons were defined as ≥2 μm in minimal diameter and DLG positive, and branches were defined as HRP-positive processes with ≥ two type 1 boutons. As reported previously, *dfmr1* null branch number was significantly increased compared with genetic controls (normalized *w^1118^*, 1.0±0.067; *dfmr1^50M^*, 1.64±0.086, *P*<0.01; Fig. 6A,B). Likewise, the number of type 1 synaptic boutons was similarly increased in the *dfmr1* nulls compared with controls (*w^1118^*, 1.0±0.069; *dfmr1^50M^*, 1.72±0.078, *P*<0.01; Fig. 6A,C). We first attempted to mimic *dfmr1* null phenotypes by inducing Dlp elevation at otherwise wild-type NMJs using postsynaptic driver 24B-GAL4, in order to assign causality to synaptic architecture defects. We confirmed Dlp overexpression by analyzing Dlp fluorescence intensity at the NMJ (normalized *w^1118^*, 1.0±0.10; 24B × UAS-*Dlp*, 3.76±0.37, *P*<0.001; supplementary material Fig. S2A,B). Supporting our hypothesis, Dlp overexpression mirrored *dfmr1* null synaptic structure phenotypes, including both increased branch number (normalized 24B × *w^1118^*, 1.0±0.05; 24B × UAS-*Dlp*, 1.62±0.09, *P*<0.001; Fig. 6A,B) and synaptic bouton number (24B × *w^1118^*, 1.0±0.044; 24B × UAS-*Dlp*, 1.41±0.056, *P*<0.001; Fig. 6A,C). Although we did not analyze Sdc overexpression, a previous study has shown that it causes a similar change in NMJ architecture (Johnson et al., 2006). These data suggest that HSPG overexpression is sufficient to produce the synapse overelaboration characterizing *dfmr1* null NMJs.

We next analyzed the consequences of heterozygous genetic reduction of *dlp* alone and in tandem with *sdc* in the *dfmr1* null background. We first confirmed that the heterozygotes express reduced Dlp protein levels (normalized *w^1118^*, 1.0±0.048; *dlp^A187^*/+, 0.65±0.054, *P*<0.001; supplementary material Fig. S2C,D). As predicted by the above Dlp overexpression results, genetic reduction of Dlp alone significantly reduced synaptic overelaboration in the *dfmr1* null as normalized to *w^1118^* genetic control, both in branch number (*dfmr1^50M^*, 1.64±0.081; *dlp^A187^*, *dfmr1^50M^*/*dfmr1^50M^*, 1.17±0.066, *P*<0.01; Fig. 6A,B) and bouton number (*dfmr1^50M^*, 1.72±0.078; *dlp^A187^*, *dfmr1^50M^*/*dfmr1^50M^*, 1.23±0.049, *P*<0.01; Fig. 6A,C). Likewise, genetic reduction of both Dlp and Sdc HSPGs in tandem similarly reduced synaptic branch number (*dfmr1^50M^*, 1.64±0.081; *sdc^23^*/+;*dlp^A187^*, *dfmr1^50M^*/*dfmr1^50M^*, 1.26±0.08, *P*<0.01; Fig. 6A,B) and bouton number (*dfmr1*^50M^, 1.72±0.078; *sdc^23^*/+; *dlp^A187^*, *dfmr1^50M^*/*dfmr1^50M^*, 1.24±0.062, *P*<0.01; Fig. 6A,C). The restored synaptic architecture of the double heterozygous mutants in the *dfmr1* null background was not significantly different from the *w^1118^* genetic control. Additional controls are shown in supplementary material Fig. S3, for branching (normalized *w^1118^*, 1.0±0.067; UAS-*Dlp*/+, 1.0±0.064; *dlp^A187^*/+, 1.08±0.09; *sdc^23^*/+; *dlp^A187^*/+, 1.04±0.11, *P*>0.05; supplementary material Fig. S3A,B) and bouton number (normalized *w^1118^*, 1.0±0.069; UAS-*Dlp*/+, 1.08±0.045; *dlp^A187^*/+, 1.05±0.074; *sdc^23^*/+; *dlp^A187^*/+, 1.07±0.065, *P*>0.05; supplementary material Fig. S3C). These results show that Dlp elevation in the *dfmr1* null accounts for synapse architecture defects in the FXS disease state condition.

### Genetically reducing HSPG levels in *dfmr1* nulls restores synaptic function

**Fig. 7.**
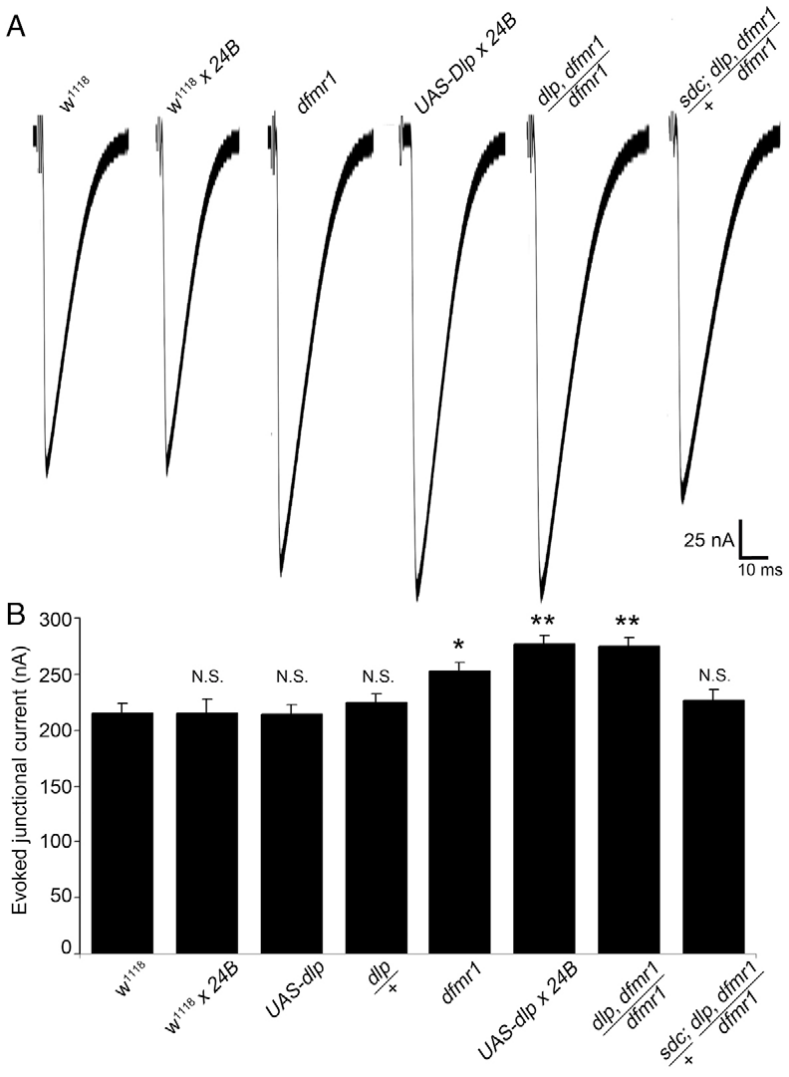
**Restoration of *dfmr1* null synaptic functional defect by HSPG reduction.** (A) Representative excitatory junctional current (EJC) traces from the following six genotypes: genetic control (*w^1118^*), *w^1118^* crossed to postsynaptic driver (24B-GAL4), homozygous *dfmr1*^50M^ null, 24B-GAL4 driving UAS-Dlp, heterozygous *dlp^A187^*/+ recombined into the *dfmr1* null background (*dlp^A18^*, *dfmr1^50M^*/*dfmr1^50M^*) and double heterozygous *dlp^A187^*/+ and *sdc^23^*/+ in *dfmr1* null background (*sdc^23^*/+; *dlp^A187^*, *dfmr1^50M^*/*dfmr1^50M^*). The nerve was stimulated in 1.0 mM external Ca^2+^ and TEVC records (−60 mV holding potential) made from muscle 6 in segment A3. Each trace is averaged from ten consecutive evoked EJC recordings. (B) Quantified mean EJC amplitudes (nA) for the six genotypes shown. Sample sizes are ≥8 animals and individual NMJ terminals. Statistical significance shown as *P<0.05, **P<0.01 and not significant (N.S.).

We have previously shown that NMJ synaptic transmission strength is significantly elevated in *dfmr1* null mutants (Zhang et al., 2001). Consistently, we have recently shown that HSPG co-receptors acting as potent regulators of trans-synaptic signaling also strongly modulate synaptic function (Dani et al., 2012). We therefore hypothesized that the observed functional change in *dfmr1* nulls might also be driven by HSPG elevation (Fig. 1). To test this hypothesis, we assayed animals with two-electrode voltage-clamp (TEVC) electrophysiology to compare excitatory junctional current (EJC) amplitudes. Representative records show ten averaged nerve-stimulation-evoked EJC responses (1.0 mM extracellular Ca^2+^) for all tested genotypes shown in Fig. 7A, with amplitude quantification shown in Fig. 7B. Consistent with previous reports, EJC amplitudes were significantly elevated by ∼20% in *dfmr1* nulls compared with controls (*w^1118^*, 215.38±8.31 nA; *dfmr1^50M^*, 252.89±7.91 nA, *P*<0.05; Fig. 7A,B). Dlp overexpression with the postsynaptic 24B-GAL4 driver resulted in a significant elevation in synaptic transmission strength as compared with the driver-alone control (*w^1118^* × *24BGAL4,* 215.36±12.04 nA; *UAS-Dlp*/+, 213.95±8.84 nA; *UAS-Dlp* × *24B-GAL4*, 276.47±8.18 nA, *P*<0.01; Fig. 7A,B). These data suggest that Dlp overexpression is sufficient to produce the synapse strengthening that is observed in *dfmr1* null NMJs.

We next examined the consequences of heterozygous genetic reduction of Dlp alone, as well as of Dlp and Sdc in combination, in the *dfmr1* null background. Compared with the elevated EJC amplitude of *dfmr1* nulls (252.89±7.91 nA, *P*<0.01, *n*=9; Fig. 7A,B), genetic reduction of Dlp alone (*dlp^A187^*, *dfmr1^50M^*/*dfmr1^50M^*) did not effectively restore transmission, as compared with genetic controls (*w^1118^*, 215.38±8.31 nA; *dlp^A187^*/+, 224.81±7.53 nA; *dlp^A187^*, *dfmr1^50M^*/*dfmr1^50M^*, 273.08±11.29 nA, *P*<0.01; Fig. 7A,B). We therefore next measured transmission strength with combinatorial reduction of both Dlp and Sdc HSPGs in the *dfmr1* null background. We found that correction of both HSPGs together fully restored EJC amplitudes to control levels (*w^1118^*, 215.38±8.31 nA; *sdc^23^*/+; *dlp^A187^*, *dfmr1^50M^*/*dfmr1^50M^*, 216.89±9.24 nA, *P*>0.05; Fig. 7A,B). Under this condition, there was no significant difference between triple-mutant animals and controls. These data reveal that reduction of both HSPG co-receptors is necessary and sufficient to correct *dfmr1* null neurotransmission back to control levels.

### Genetically reducing HSPG levels in *dfmr1* nulls restores normal trans-synaptic signaling

Based on the above studies, our working hypothesis was that FMRP loss causes HSPG elevation to alter trans-synaptic signal pathways regulating synaptic structure and function. To test this proposed cascade, we directly assayed whether introducing the heterozygous HSPG mutations into the *dfmr1* null background restores Wg and Jeb signaling. By removing one copy of both *dlp* and *sdc* in tandem, we aimed to suppress the synaptic HSPG elevation caused by loss of FMRP (Fig. 1). To assess the success of this genetic manipulation, we first analyzed NMJ expression levels of Dlp and Sdc in the triple-mutant condition (supplementary material Fig. S4). As expected, both Dlp (normalized *w^1118^*, 1.0±0.044; *sdc^23^*/+; *dlp^A187^*, *dfmr1^50M^*/*dfmr1^50M^*, 0.89±0.043, *P*>0.05; supplementary material Fig. S4A,B) and Sdc (*w^1118^*, 1.0±0.066; *sdc^23^*/+; *dlp^A187^*, *dfmr1^50M^*/*dfmr1^50M^*, 1.04±0.099, *P*>0.05; supplementary material Fig. S4C,D) levels were reduced in the heterozygous condition, and were no longer significantly different from controls, indicating that the *dfmr1* null phenotypes were fully suppressed in the triple-mutant condition.

We next assayed the trans-synaptic signaling ligands in the extracellular synaptomatrix. In two different *dfmr1* nulls, Wg levels were significantly elevated at the NMJ synapse (Fig. 2). In the triple-mutant condition, Wg was no longer elevated and levels were not significantly different from control (*w^1118^*, 1.0±0.081; *sdc^23^*/+;*dlp^A187^*, *dfmr1^50M^*/*dfmr1^50M^*, 0.88±0.12, *P*>0.05; supplementary material Fig. S5A,B). Conversely, in the two *dfmr1* nulls, Jeb levels were significantly reduced at the NMJ (Fig. 4A,B). In the triple-mutant condition, synaptic Jeb expression was restored back towards normal and was no longer significantly different from controls (*w^1118^*, 1.0±0.059; *sdc^23^*/+; *dlp^A187^*, *dfmr1^50M^*/*dfmr1^50M^*, 0.89±0.064, *P*>0.05; supplementary material Fig. S5C,D). We finally assayed downstream nuclear signaling for both pathways. For the Wg pathway, Fz2-C nuclear localization was strongly reduced in two different *dfmr1* nulls (Fig. 3). In the triple-mutant condition, Fz2-C puncta number in the muscle nuclei was restored back towards normal and was no longer significantly different from controls (*w^1118^*, 1.0±0.099; *sdc^23^*/+;*dlp^A187^*, *dfmr1^50M^*/*dfmr1^50M^*, 0.71±0.16, *P*>0.05; supplementary material Fig. S6A,B). For the Jeb pathway, dpErk nuclear localization was likewise reduced in the two *dfmr1* nulls (Fig. 4C,D). In the triple-mutant condition, nuclear dpErk levels were also restored to the control situation (fluorescence intensity: *w^1118^*, 1.0±0.027; *sdc^23^*/+; *dlp^A187^*, *dfmr1^50M^*/*dfmr1^50M^*, 0.92±0.035, *P*>0.05; supplementary material Fig. S6C,D). These findings suggest that the HSPG co-receptor elevation in *dfmr1* null mutants is causal for the changes in both Wg and Jeb trans-synaptic signaling and transduction pathways, providing a mechanism to explain the excess synaptic architecture and function in this FXS disease model.

## DISCUSSION

FXS is widely considered a disease state arising from synaptic dysfunction (Bassell and Warren, 2008; Pfeiffer and Huber, 2009; Bhakar et al., 2012), with pre- and postsynaptic defects well characterized in the *Drosophila* disease model (Zhang et al., 2001; Pan et al., 2004; Gatto and Broadie, 2011; Tessier and Broadie, 2012). There has been much work documenting FXS phenotypes in humans as well as in animal models (Rudelli et al., 1985; Hinton et al., 1991; Gatto and Broadie, 2011; Gross et al., 2012; Tessier and Broadie, 2012), but there has been less progress on mechanistic underpinnings. Our FXS focus has shifted to the extracellular synaptomatrix owing to identification of pharmacological and genetic interactions between FMRP and secreted MMPs (Siller and Broadie, 2011), a mechanism that is conserved in mammals (Bilousova et al., 2009; Siller and Broadie, 2012). Other work in our laboratory has also recently highlighted the importance of the synaptomatrix in synaptogenesis (Dani and Broadie, 2012), particularly the roles of membrane-anchored HSPGs as co-receptors regulating trans-synaptic signaling. Importantly, recent work has also shown that FMRP binds HSPG mRNAs (Darnell et al., 2011), thereby presumably repressing translation. Based on these multiple lines of evidence, we hypothesized that the FMRP-MMP-HSPG intersection provides a coordinate mechanism for the pre- and postsynaptic defects characterizing the FXS disease state, with trans-synaptic signaling orchestrating synapse maturation across the synaptic cleft (McCabe et al., 2003; Korkut and Budnik, 2009; Ball et al., 2010; Budnik and Salinas, 2011; Rohrbough et al., 2013).

In testing this hypothesis, we discovered dramatic upregulation of GPI-anchored glypican Dlp and transmembrane Sdc HSPGs at *dfmr1* null NMJ synapses. Indeed, these are among the largest synaptic molecular changes reported in the *Drosophila* FXS disease model (Tessier and Broadie, 2012). Importantly, HSPGs have been shown to play key roles in synaptic development. For example, the mammalian HSPG Agrin has long been known to regulate acetylcholine receptors (Parkhomovskiy et al., 2000), interconnected with a glycan network modulating trans-synaptic signaling (Kleene and Schachner, 2004). In *Drosophila*, Dlp, Sdc and Perlecan HSPGs mediate axon guidance, synapse formation and trans-synaptic signaling (Yamaguchi, 2001; Lee and Chien, 2004; Van Vactor et al., 2006; Dani et al., 2012; Kamimura et al., 2013). Previous work on *dlp* mutants has reported elevated neurotransmission (Johnson et al., 2006), paradoxically similar to the Dlp overexpression phenotype shown here. However, the previous study did not show Dlp overexpression electrophysiological data, although it did show increased active zone areas consistent with strengthened neurotransmission. The same study reported that Dlp overexpression decreased bouton number on muscle 6/7, which differs from our finding of increased bouton number on muscle 4. Because HSPG co-receptors regulate trans-synaptic signaling (Dani et al., 2012), we next tested *dfmr1* mutants for changes in three established pathways at the *Drosophila* NMJ. We found there to be strong alterations in both Wg and Jeb pathways, with anterograde signaling being downregulated in both cases. In contrast, we found no change in the retrograde BMP Gbb pathway, suggesting that FMRP plays specific roles in modulating anterograde trans-synaptic signaling during synaptogenesis.

The defect in Jeb signaling seems to be simple to understand, with decreased synaptomatrix ligand abundance coupled to decreased dpERK nuclear localization (Rohrbough et al., 2013). However, there is no known link to HSPG co-receptor regulation. We have shown that Jeb signaling is regulated by another synaptomatrix glycan mechanism (Rohrbough and Broadie, 2010), providing a clear precedent for this level of regulation. In contrast, the Wnt pathway exhibits an inverse relationship between Wg ligand abundance (elevated) and Fz2-C nuclear signaling (reduced). This apparent contradiction is explained by the dual activity of the Dlp co-receptor, which stabilizes extracellular Wg to retain it at the membrane (Han et al., 2005; Yan and Lin, 2009), but also competes with the Fz2 receptor (Yan et al., 2009). This ‘exchange-factor mechanism’ is competitively dependent on the ratio of Dlp co-receptor to Fz2 receptor, with a higher ratio causing more Wg to be competed away from Fz2. Indeed, we recently demonstrated the same elevated Wg surface retention coupled to decreased downstream Fz2-C signaling in an independent HSPG regulative mechanism at the *Drosophila* NMJ (Dani et al., 2012). In the *dfmr1* null synapse, we suggest that highly elevated Dlp traps Wg, thereby preventing it from binding Fz2 to initiate signaling.

Dysregulation of the Wg nuclear import pathway (FNI) provides a plausible mechanism to explain synapse development defects underlying the FXS disease state, with established roles in activity-dependent modulation of synaptic morphogenesis and neurotransmission (Korkut and Budnik, 2009). FXS has long been associated with defects in activity-dependent architectural modulation, including postsynaptic spine formation, synapse pruning and functional plasticity (Mercaldo et al., 2009; Pfeiffer and Huber, 2009; Tessier and Broadie, 2008). Although it is surely not the only player, aberrant Wg signaling could play a part in these deficiencies. Importantly, recent work has shown that the FNI pathway is involved in shuttling large RNA granules out of the postsynaptic nucleus (Speese et al., 2012), providing a potential intersection with the FMRP RNA transport mechanism (Bassell and Warren, 2008; Kao et al., 2010). However, the Wg FNI pathway is not the only Wnt signaling at the *Drosophila* NMJ, with other outputs including the canonical, divergent canonical and planar cell polarity pathways (Korkut and Budnik, 2009), which could be dysregulated in *dfmr1* nulls. For example, a divergent canonical retrograde pathway proceeds through GSK3β (Shaggy) to alter microtubule assembly (Miech et al., 2008), and the FXS disease state is linked to dysregulated GSK3β (Min et al., 2009; Yuskaitis et al., 2010; Mines and Jope, 2011) and microtubule stability misregulation via *Drosophila* Futsch/mammalian MAP1B (Zhang et al., 2001; Lu et al., 2004; Yao et al., 2011). Moreover, it was recently shown that the secreted HSPG Perlecan (*Drosophila Trol*) regulates bidirectional Wnt signaling to affect *Drosophila* NMJ structure and/or function, via anterograde FNI and retrograde divergent canonical pathways (Kamimura et al., 2013). It is also important to note that previous studies have shown that a reduction in the FNI pathway, due to decreased Fz2-C trafficking to the nucleus, leads to decreased NMJ bouton number (Mathew et al., 2005; Ataman et al., 2006). Future work is needed to fully understand connections between FMRP, HSPGs, the multiple Wnt signaling pathways and the established defects in the synaptic microtubule cytoskeleton in the FXS disease state.

Adding to the complications of FXS trans-synaptic signaling regulation, we show here that two trans-synaptic signaling pathways are suppressed in parallel: the Wg and Jeb pathways. Possibly even more promising for clinical relevance, we have just recently established that the Jeb signaling functions as a repressor of neurotransmission strength at the *Drosophila* NMJ, with *jeb* and *alk* mutants presenting increased evoked synaptic transmission (Rohrbough et al., 2013). Consistently, loss of FMRP leads to increased EJC amplitudes, which could be due, at least partially, to misregulated Jeb-Alk signaling. Importantly, we have shown that *dfmr1* null neurotransmission defects are due to a combination of pre- and postsynaptic changes (Zhang et al., 2001; Pan and Broadie, 2007; Pan et al., 2008; Repicky and Broadie, 2009), and that there is a non-cell-autonomous requirement for FMRP in the regulation of functional changes in the synaptic vesicle (SV) cycle underlying neurotransmission strength (Gatto and Broadie, 2008). Additionally, *jeb* and *alk* mutants exhibit synaptic structural changes consistent with this FMRP interaction, including a larger NMJ area and synaptic bouton maturation defects (Rohrbough et al., 2013), which are markedly similar to the structural overelaboration phenotypes of the FXS disease state (Gatto and Broadie, 2011; Tessier and Broadie, 2012). These data together suggest that altered Jeb-Alk trans-synaptic signaling plays a role in the synaptic dysfunction characterizing the *dfmr1* null. We propose that Wg and Jeb signaling defects likely interact, in synergistic and/or antagonistic ways, to influence the combined pre- and postsynaptic alterations characterizing the FXS disease state.

Although trans-synaptic signaling pathways, and in particular both Wnt and Jeb-Alk pathways, have been proposed to be involved in the manifestation of a number of neurological disorders (Okerlund and Cheyette, 2011; Smith and Sadee, 2011; Weiss et al., 2012), we provide here the first evidence that aberrant trans-synaptic signaling is causally involved in an FXS disease model. We propose a mechanism in which FMRP acts to regulate trans-synaptic ligands by depressing expression of membrane-anchored HSPG co-receptors. HSPG overexpression alone is sufficient to cause both synaptic structure and function defects characterizing the FXS disease state. Increasing HSPG abundance in the postsynaptic cell was enough to increase the number of presynaptic branches and synaptic boutons, as well as elevate neurotransmission. Correlation with these well-established *dfmr1* null synaptic phenotypes suggested that HSPG elevation could be a causal mechanism. Conclusively, reversing HSPG overexpression in the *dfmr1* null is sufficient to correct Wnt and Jeb signaling, and to restore normal synaptic structure and function. Because there is no dosage compensation, HSPG heterozygosity offsets the elevation caused by loss of *dfmr1*. Correcting both Dlp and Sdc HSPGs in the *dfmr1* background restored Wg and Jeb signaling to control levels. Correcting Dlp levels by itself restored synaptic architecture, but both Dlp and Sdc had to be corrected to restore normal neurotransmission in *dfmr1* null synapses. Taken together, these results from the *Drosophila* FXS disease model provide exciting new insights into the mechanisms of synaptic phenotypes caused by the loss of FMRP, and promising avenues for new therapeutic treatment strategies.

## MATERIALS AND METHODS

### Drosophila genetics

Two *dfmr1* null alleles (*dfmr1^50M^* and *dfmr1^2^*) (Zhang et al., 2001; Dockendorff et al., 2002) were used, with homozygous mutants being selected using GFP balancer chromosomes and compared with the *w^1118^* genetic background control. For overexpression studies, UAS-*dlp* and 24B-GAL4 driver lines (Brand and Perrimon, 1993) were crossed for elevated postsynaptic expression. Double mutants were generated by recombining the *dlp^A187^* null allele (Han et al., 2004) onto the *dfmr1^50M^* null chromosome (*dlp^A187^*, *dfmr1^50M^*/TM6Hu-GFP). This line was crossed to *dfmr1^50M^*/TM6Tb-GFP, to generate *dlp^A187^*, *dfmr1^50M^*^/^/*dfmr1^50M^* (50% reduced Dlp in the *dfmr1* null background). The *sdc^23^* null allele (Steigemann et al., 2004) was crossed to *dfmr1^50M^*/TM6Tb-GFP to generate *sdc^23^*/Cyo-GFP; *dfmr1^50M^*/TM6Tb-GFP. The two lines were crossed to create *sdc^23^*/+; *dlp^A187^*, *dfmr1^50M^*/*dfmr1^50M^* flies (50% reduced Sdc and Dlp in the *dfmr1* null background).

### Immunocytochemistry

Wandering third instars were fixed using Bouin’s fixative for 30 minutes (Dlp) or 4% paraformaldehyde for 10 minutes (everything else). Preparations were rinsed 3× with PBS and processed without detergent for extracellular labeling (Dlp, Sdc, Wg, Gbb, Jeb), or with 0.2% Triton X-100 detergent for intracellular labeling (dFz-C, pMad, dpErk, DLG), with anti-HRP labeling neuronal membranes (Rushton et al., 2009; Dani et al., 2012). Preparations were incubated with primary antibodies overnight at 4°C, followed by secondary antibodies for 4 hours at room temperature (RT). Primary antibodies included: mouse anti-Dlp [13G8, 1:4; Developmental Studies Hybridoma Bank (DSHB)], rabbit anti-Sdc (1:200) (Spring et al., 1994), mouse anti-Wg (4D4, 1:2; DSHB), rabbit anti-Gbb (1:100) (Dani et al., 2012), guinea pig anti-Jeb (1:1000) (Rohrbough and Broadie, 2010), rabbit anti-dFz-C (1:500) (Mathew et al., 2005), rabbit anti-pMAD (1:1000) (Persson et al., 1998), mouse anti-dpERK (M8159, 1:1000; Sigma-Aldrich) (Rohrbough et al., 2013), mouse anti-DLG (DLG1, 1:200; DSHB). Secondary antibodies included: Alexa-Fluor-488-conjugated goat anti-mouse IgG (1:200), Alexa-Fluor-488-conjugated goat anti-rabbit IgG (1:250) and Alexa-Fluor-488-conjugated goat anti-guinea pig IgG (all from Invitrogen-Molecular Probes). Neuronal presynaptic terminals were co-labeled using anti-HRP directly conjugated to Cy3 (1:100; Jackson ImmunoResearch Laboratories, Inc.). Nuclei were labeled using propidium iodide (PI; 1:1000; Sigma-Aldrich) or DRAQ5 (1:1000; Cell Signaling Technology) to mark DNA following RNase incubation (Dani et al., 2012). Preparations were mounted in Fluoromount G (EMS, Hatfield, PA) and fluorescent images collected with an upright Zeiss LSM 510 META laser-scanning confocal microscope.

### Imaging quantification

Analyses were done on wandering third instar lateral longitudinal muscle NMJs (4, 6/7) and corresponding motor neuron cell bodies in abdominal segment 3 (combined left and right hemisegments for each; *n*=1). All expression analyses were done using NIH ImageJ software with the threshold function outlining HRP-labeled NMJs and PI-labeled nuclei. All figure images are maximum projection Z-stacks, with intensity measured as maximum pixel intensity. Intensities and areas were measured as the outlined signal of interest area over the total HRP-labeled NMJ area to normalize for synaptic terminal size. A threshold of two standard deviations below the control mean was used to determine threshold boundaries. NMJ structural parameters were quantified as previously described (Gatto and Broadie, 2008; Coffee et al., 2010). Briefly, NMJs were co-labeled with presynaptic anti-HRP and postsynaptic anti-DLG in muscle 4 NMJ from segment A3. Type 1 boutons were defined as >2 μm in diameter and DLG-positive. Synaptic branches were defined as a terminal axonal projection contained ≥ two type 1 boutons. ImageJ software was used to threshold fluorescence intensity and count synaptic boutons and branches.

### Electrophysiology

TEVC records were made from the wandering third instar as previously described (Beumer et al., 1999; Dani and Broadie, 2012). Briefly, dissected animals were secured on sylgard-coated coverslips with surgical glue (liquid suture), and segmental nerves were cut near the ventral nerve cord. Recording was performed in 128 mM NaCl, 2 mM KCl, 4 mM MgCl_2_, 1 mM CaCl_2_, 70 mM sucrose and 5 mM Hepes. Recording electrodes were filled with 3 M KCl and had resistances of >10 MΩ. Evoked EJC recordings were made from the voltage-clamped (*V_hold_*=−60 mV) muscle 6 in segment A3 with a TEVC amplifier (Axoclamp 200B; MDS Analytical Technologies). The cut segmental nerve was stimulated with a glass suction electrode at a suprathreshold voltage level (50% above baseline threshold value) for 0.2–0.5 ms duration. Records were made with 0.2 Hz nerve stimulation with episodic acquisition and analyzed with Clampex software (version 7.0; Axon Instruments).

## Supplementary Material

Supplementary Material
