# Supplementary material for: Fragile X mental retardation protein regulates trans-synaptic signaling in Drosophila
Source: Dis Model Mech. 2013 Sep 5;6(6):1400–13. doi: 10.1242/dmm.012229 (PMC3820263; doi:10.1242/dmm.012229)
Supplement: Supplementary Material [file supp_6_6_1400__index.html]

Fragile X mental retardation protein regulates trans-synaptic signaling in Drosophila — Fragile X mental retardation protein regulates trans-synaptic signaling in Drosophila — Supplementary Material 

# Fragile X mental retardation protein regulates trans-synaptic signaling in *Drosophila*

## DMM012229 Supplementary Material

**Files in this Data Supplement:**

- **Supplementary Material PDF**
