# Supplementary material for: Fragile X mental retardation protein regulates trans-synaptic signaling in Drosophila
Source: Dis Model Mech. 2013 Sep 5;6(6):1400–13. doi: 10.1242/dmm.012229 (PMC3820263; doi:10.1242/dmm.012229)
Supplement: Supplementary Material [file supp_012229_DMM012229.pdf]

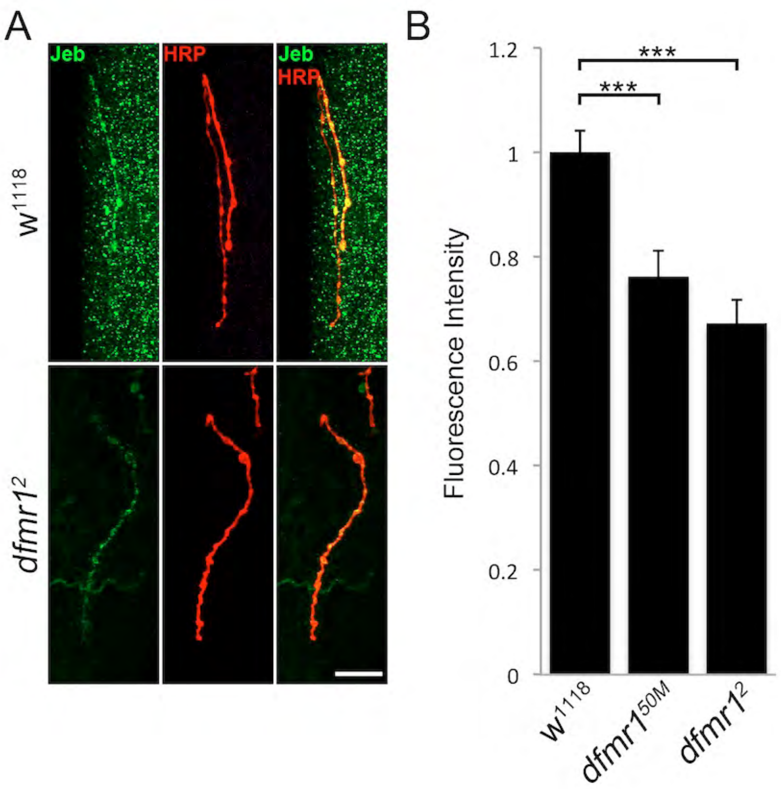

Supplementary material Fig. S1. Reduced Jeb levels in the *dfmr1* null muscle. (A) Representative NMJ images co-labeled with presynaptic marker (HRP, red) and anti-Jelly Belly (Jeb, green) in control (w^{1118}) and *dfmr1* null ($dfmr1^{50M}$) wandering third instar muscle 4. Scale bar: 15µm. (B) Quantification of Jeb muscle surface intensity away from the NMJ domain in two *dfmr1* null alleles ($dfmr1^{50M}$, $dfmr1^{2}$) normalized to the genetic control (w^{1118}). Sample sizes are ≥9 animals and ≥18 muscles for each of the three genotypes. Statistical significance shown as ****P*<0.001.

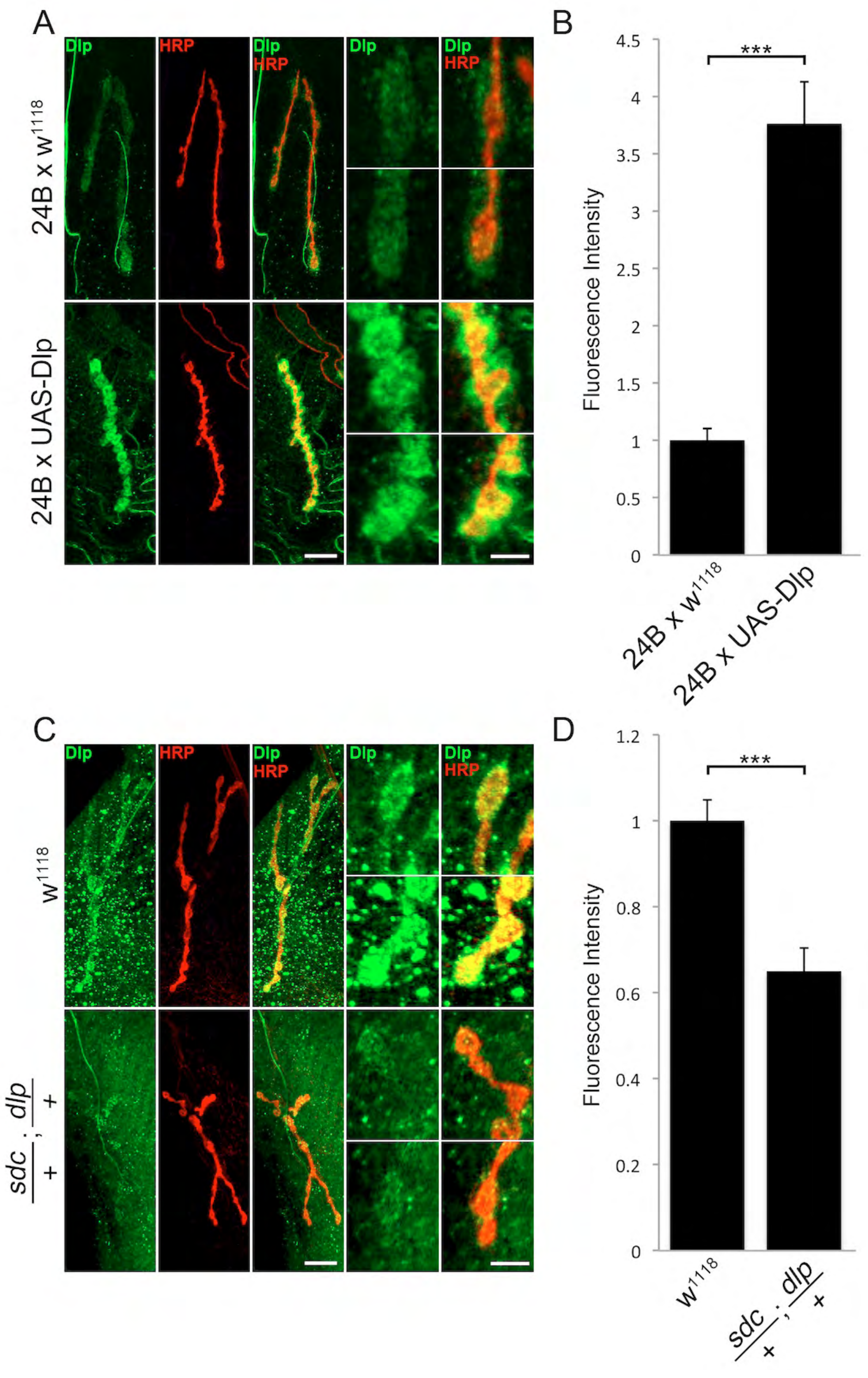

Supplementary material Fig. S2. Quantification of Dlp levels with genetic manipulations. (A) Representative NMJ images co-labeled with presynaptic marker anti-horseradish peroxidase (HRP, red) and anti-Dally-like Protein (Dlp, green) in control (w^{1118}) and postsynaptic Dlp overexpression (*24B* x UAS-*Dlp*) conditions. Scale bars: 15µm and 5 µm. (B) Quantification of Dlp intensity normalized to genetic control (w^{1118}). Sample size is ≥3 animals and ≥6 NMJs for each genotype. (C) Representative NMJ images in control and double heterozygous dlp^{A187}/+; sdc^{23}/+ conditions. Scale bars: 15µm and 5 µm. (D) Quantification of Dlp intensity normalized to genetic control (w^{1118}). Sample size is ≥8 animals and ≥16 NMJs for each genotype. Statistical significance shown as ****P*<0.001.

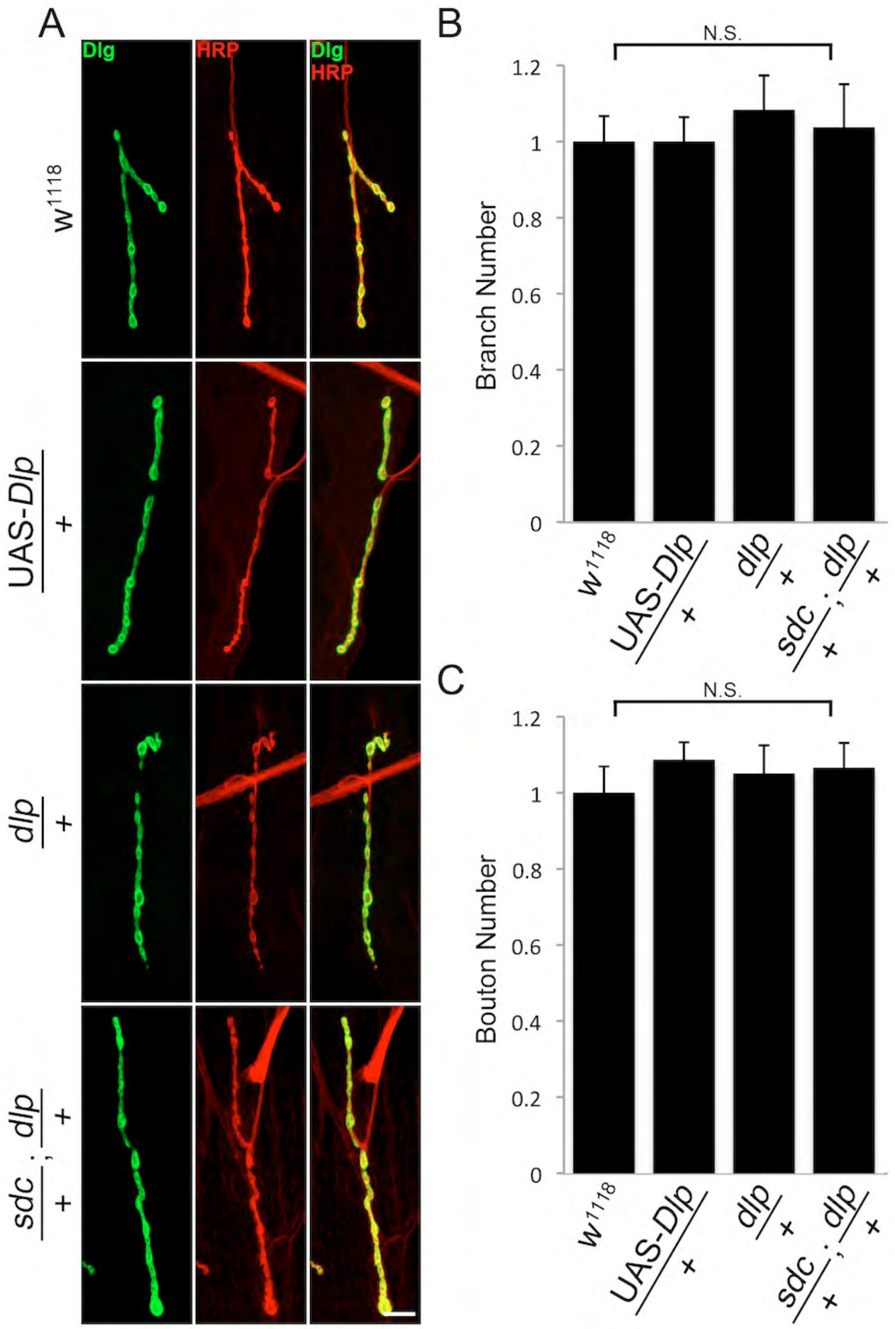

Supplementary material Fig. S3. Genetic controls for NMJ synaptic architecture studies. (A) Representative muscle 4 NMJ images co-labeled with presynaptic marker anti-horseradish peroxidase (HRP, red) and postsynaptic marker anti-Discs Large (DLG, green) in control (*w*^{1118}), *w*^{1118} crossed to UAS-Dlp, heterozygous *dlp*^{A187}/+, and double heterozygous *dlp*^{A187}/+; *sdc*^{23}/+. Scale bar: 15µm. Quantification of NMJ branch (B) and type 1 bouton (C) numbers. Sample size is ≥6 animals and ≥12 NMJs for each genotype. Statistical significance shown as N.S. (P>0.05).

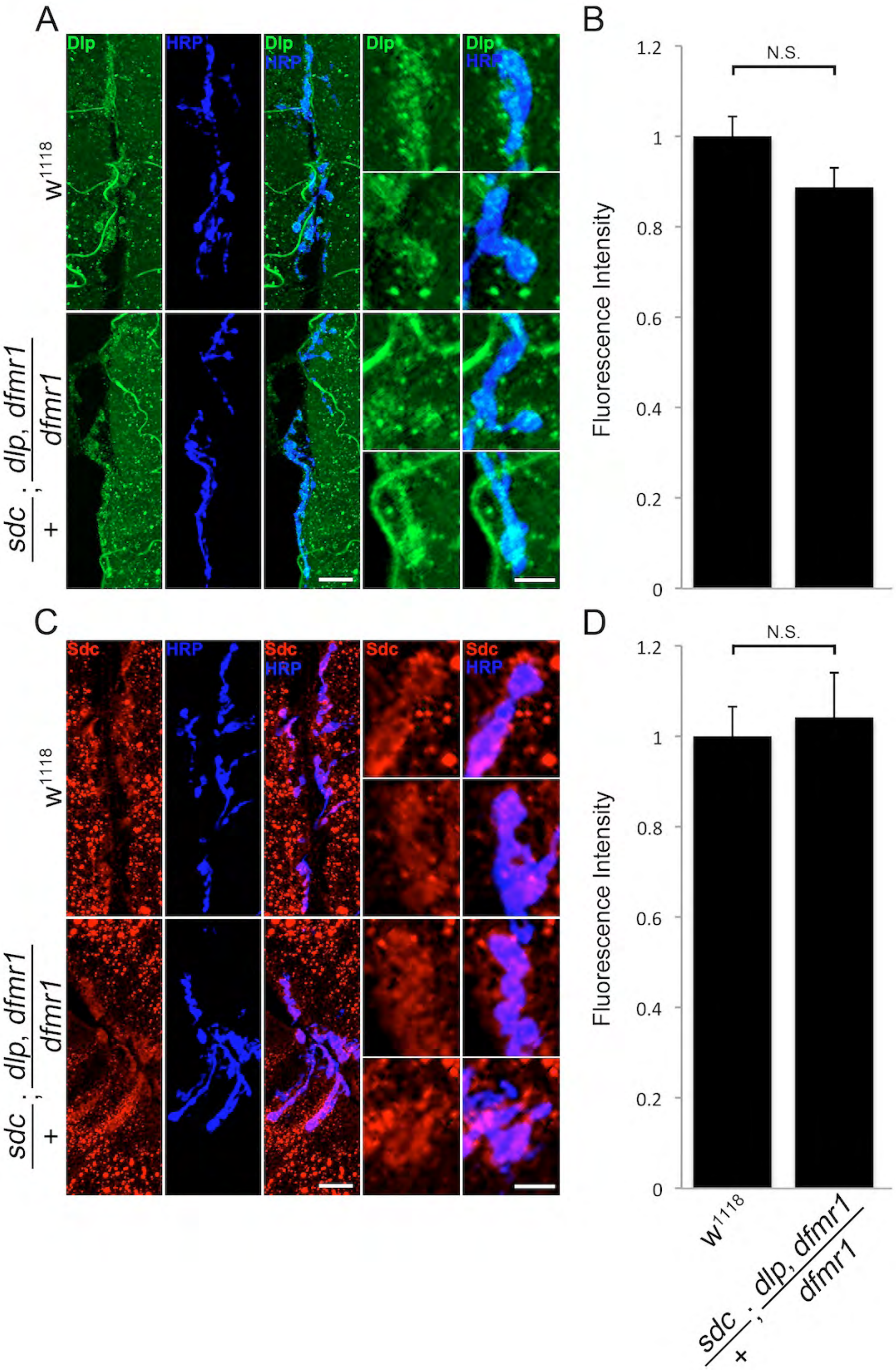

Supplementary material Fig. S4. Synaptic HSPG levels in the triple mutant condition. (A) Representative NMJ images co-labeled with anti-HRP (red) and anti-Dlp (green) in genetic control (*w^1118^*) and *sdc^23^*/+; *dlp^A187^*, *dfmr1^50M^*/*dfmr1^50M^* triple mutant condition. Scale bars: 25µm and 5µm. (B) Quantification of Dlp intensity normalized to control. Sample size is ≥8 animals and ≥16 NMJs for genotype. (C) Representative NMJ images co-labeled with anti-HRP (red) and anti-Syndecan (Sdc, green) in control and triple mutant. Scale bars: 25µm and 5µm. (D) Quantification of Sdc intensity normalized to control. Sample sizes are ≥8 animals and ≥16 NMJs for each genotype. Statistical significance shown as N.S. (*P*>0.05).

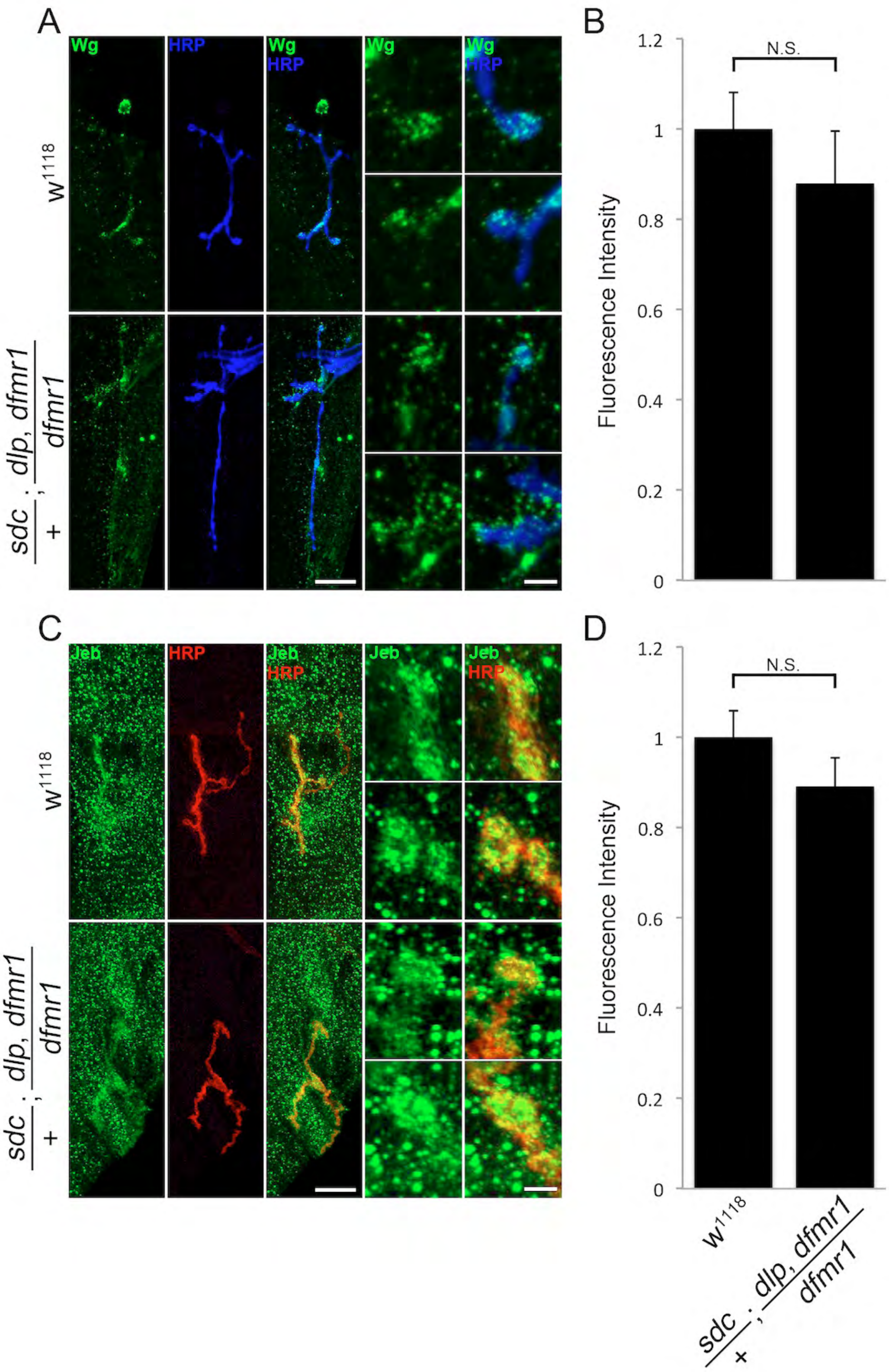

Supplementary material Fig. S5. Wg and Jeb ligand levels in the triple mutant condition. (A) Representative NMJ images co-labeled with anti-HRP (red) and anti-Wingless (Wg, green) in control (*w*^{1118}) and *sdc*^{23}/+; *dlp*^{A187}, *dfmr1*^{50M}/*dfmr1*^{50M} triple mutant conditions. Scale bars: 15μm and 5 μm. (B) Quantification of Wg intensity normalized to control. Sample size is ≥8 animals and ≥15 NMJs for each genotype. (C) Representative NMJ images co-labeled with anti- HRP (red) and anti-Jelly Belly (Jeb, green) in *w*^{1118} and triple mutant conditions. Scale bars: 15μm and 5μm. (D) Quantification of Jeb intensity normalized to control. Sample sizes are ≥8 animals and ≥16 NMJs for each genotype. Statistical significance shown as N.S. (*P*>0.05).

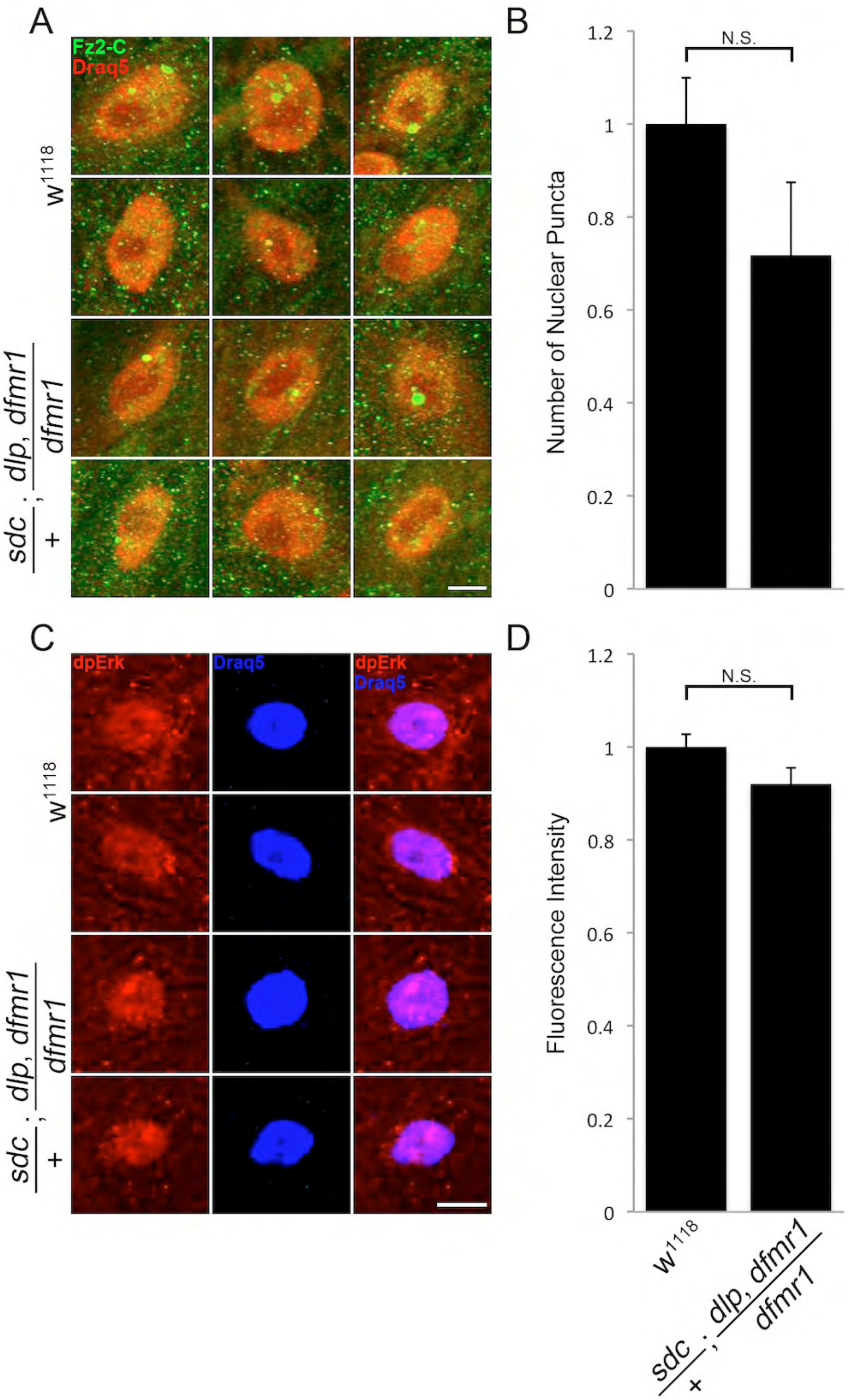

Supplementary material Fig. S6. Fz2C- and dpERK nuclear localization in the triple mutant condition. (A) Representative images of wandering third instar muscle 4 nuclei co-labeled with nuclear marker Draq5 (red) and anti-Frizzled C-terminus (Fz2-C, green) in control (*w*^{1118}) and *sdc*^{23}/+; *dlp*^{A187}, *dfmr1*^{50M}/*dfmr1*^{50M} triple mutant conditions. Scale bar: 5μm. (B) Quantification of Fz2-C nuclear localization, measured as Fz2-C puncta number, normalized to genetic control. Sample sizes are ≥3 animals and ≥6 muscles for each genotype. (C) Representative images of muscle nuclei co-labeled with nuclear marker Draq5 (red) and anti-diphosphorylated extracellular signal regulated kinase (dpERK, green) in control and triple mutant. Scale bar: 5μm. (D). Quantification of dpERK nuclear intensity levels normalized to genetic control. Sample sizes are ≥8 animals and ≥15 muscles for each genotype. Statistical significance shown as N.S. (P>0.05).
